# In search of common ground: Exploring value networks at the UNFCCC climate change talks

**DOI:** 10.1017/nws.2026.10027

**Published:** 2026-04-13

**Authors:** Zack W. Almquist, Benjamin Bagozzi, Daria Blinova, Zach Brown

**Affiliations:** 1 Department of Sociology, University of Washington, USA; 2 Department of Political Science, University of Delaware, USA

**Keywords:** Climate change, values, UNFCCC, social networks, text-as-data

## Abstract

Understanding the values held by negotiating parties is central to the design and success of international climate change agreements. However, empirical understandings of these values – and the manners by which they structure negotiating countries’ value networks and interactions over time – are severely limited. In addressing this shortcoming, this paper uses keyword-assisted topic models to extract value networks for the 13 most recent Conferences of the Parties (COPs) to the United Nations Framework Convention on Climate Change (UNFCCC). It then uses network analysis tools to unpack these networks in relation to influential values, countries, and time. In doing so, it demonstrates that countries’ core climate change values (i) can be accurately recovered from COP High-level Segment (HLS) speeches and (ii) can, in turn, be used to understand the structure of negotiation networks at the UNFCCC. Analysis of the corresponding value networks for COPs 16–28 indicates that initially central values of “Fairness” and “Power” have increasingly given way to values associated with the “Environment” and “Achievement.” Thus, countries at the UNFCCC have increasingly eschewed values associated with common but differentiated responsibilities in favor of a consensus over the urgency of collectively combating climate change. These and related insights illustrate our approach’s potential for recovering and understanding value networks within climate change negotiations – a critical first step for any successful climate change agreement.

## 1. Introduction

Climate change is arguably the largest existential threat to humanity in the twenty-first century (Lorey, 2003). While aspects of climate change continue to be contested worldwide, it has galvanized major international discussions and long-term goals for humanity. This is most typified by the long-standing United Nations Framework Convention on Climate Change (UNFCCC). The UNFCCC entered into force in 1994, followed in 1995 by its first Conference of the Parties (COP). These COPs have been held annually thereafter,^1^ and have come to serve as the primary forum for multilateral negotiations over global climate change solutions. The UNFCCC’s COPs have accordingly enjoyed exceptionally high attendance (Blinova, et al., 2024), with virtually every UN-accredited nation–state now participating as (i) a Party to the UNFCCC with formal voting rights and obligations to the Convention or (ii) an Observer state that does not hold such entitlements but can nevertheless attend and participate in the UNFCCC’s COPs without a decision-making role. In these manners, climate change has become one of the major drivers of international cooperation over the last 30 years (Ferrari and Pagliari, 2021).

Even so, many have contended that the UNFCCC, and multilateral climate cooperation more generally, have underachieved in addressing the global climate change problem in a timely manner (Harris, 2022; UNEP, 2022; Leiter, 2023). While a number of contributing factors for these shortcomings have been put forward, one frequently cited contributor is the clash of values that underpins countries’ preferences and negotiation stances in this area (Bauhr, 2009; Esty and Moffa, 2012; Khrushcheva and Maltby, 2016). As such, understanding the values held by negotiating parties can be seen as central to the design and success of international climate change agreements.

This is not a new insight. Indeed, the importance of understanding attitudes, beliefs, and values has long been of interest to social scientists because these orientations have been shown to give meaning and order to society (Abelson, 1983; Swidler, 1986; Eckstein, 1988; Romney et al., 1996; Pachucki and Breiger, 2010; McLean, 2016; DiMaggio et al., 2018). Yet, with respect to multilateral climate change negotiations, empirical understandings of exactly how values underpin the UNFCCC’s high-dimensional negotiating space – and the manners by which they structure negotiating countries’ social networks and interactions over time – have proven scarce. In this work, we accordingly marry text-as-data and social network analysis techniques to uncover the value networks (Leifeld, 2013; Wang, et al., 2019) that underlie countries operating as Parties or Observers at the UNFCCC’s COPs. Given that the UNFCCC’s COPs are the preeminent venue for climate negotiations and world planning on climate change, understanding value networks in this context is among our best strategies for understanding how countries have come to approach, disagree, and concur over aspects of climate change cooperation.

In this paper, we accordingly aim to understand the undercurrents of international climate negotiation dynamics. The crux of our approach entails the generation and analysis of bipartite value networks for the 13 most recent COPs of the UNFCCC. To facilitate this analysis, we first collect a comprehensive corpus of country COP speeches and apply keyword-assisted topic models (Eshima, Imai, and Sasaki, 2024) to these speeches to extract country-year value networks for each of our 13 COPs. Network analysis of the corresponding value networks for COPs 16–28 indicates several key value shifts over the 2010–2023 period. We find clear evidence in this context that initially central values such as “Fairness” and “Power” have increasingly given way first to values of “Conformity” and “Responsibility” and later to values associated with the “Environment” and “Achievement.” These trends track closely onto collective pivots away from the Kyoto Protocol and principles of common but differentiated responsibility, subsequent international convergence over the Paris Agreement, and then ultimately a broadening (albeit by no means unanimous) acceptance of collective responsibility for climate change mitigation. These insights, along with others outlined below, illustrate the potential to recover and understand value networks within climate change negotiations, a critical first step toward a successful climate change agreement.

The remainder of this paper is structured as follows. We first advance a theoretical motivation for studying country values within the context of the UNFCCC and its COPs. We then provide further background on the UNFCCC and its COPs. This is followed by a detailed overview of our speech text sample and the Keyword Assisted Topic Model (KeyATM) approach that we use to extract values from these texts. The results from this KeyATM application, in turn, lead to our construction of value networks and corresponding network analysis. We then provide a brief discussion and conclusion.

## 2. Theoretical motivation

Addressing global challenges requires cooperative negotiation (Monheim, 2014). However, such cooperation is only possible when actors’ interests align. As international relations scholars note, the specifics of a given international cooperation circumstance undoubtedly play a role in this alignment by shaping actors’ more immediate incentives and interests (Fearon, 1998; Morrow, 1999). However, broader research from philosophy, sociology, and psychology suggests that actors’ interests are also shaped by a set of more abstract values that transcend any particular situation (Dietz, et al., 2005; Steg and de Groot, 2012). This is especially the case in the context of environmental values. To this end, intrinsic or instrumental values (e.g., values based on altruism or self-interest), are widely seen to operate in a broader sense to define actors’ behaviors and preferences in relation to environmental problems. Understandings of such environmental values have now been developed through cultural, social, economic, or universal vantages, as well as from materialist or postmaterialist viewpoints, with an eye towards actors’ external conditions and related factors (Dietz, et al., 1998; Dietz, et al., 2005; Schwartz and Bilsky, 1987; Axelrod, 1994; Inglehart, 1995). Further, while much of the above work was established at the level of the individual, subsequent research has illustrated its potential to hold at the group, community, and nation–state levels (Westing, 1996; Albin, 2003; Bouman et al., 2021; Srinivasan, et al., 2019; Tsygankov and Tsygankov, 2021; Gutiérrez-Zamora et al., 2023). As such, and in line with the influence of values on individual behaviors, there is likely to exist a diverse set of values^2^ that shape aggregate environmental attitudes (such as support for global actions) in a manner that in turn conditions the interests of national actors in international climate cooperation contexts (Stern et al., 1999). Thus, understanding values through this national lens is central to the understanding of drivers behind the design and achievement of international environmental agreements (Haas, 1989; Epstein, 2008; Sprinz and Vaahtoranta, 2017).

Given their stakes and complexity, international negotiations over climate change indeed represent an extreme case of the above dynamics (Lange et al., 2010). These negotiations involve an expansive number of national governments as formal negotiating parties – now representing virtually every country in the world. These actors, in turn, exhibit divergent political and social cultures, levels of development, contributions to CO2 emissions, and exposure to future climate change impacts. This reality necessitates careful consideration of international climate talks not simply as a complex, multifaceted phenomenon shaped by individual goals (Torstad and Saelen, 2018), but as a process in which common values are exchanged and formed through periodic interactions aimed at addressing the shared concern about the threat of climate change. To this end, an effective and coordinated response to climate change requires a unified approach with a genuine convergence of values among the actors involved. However, how much do we actually know about the nature and structure of shared values among countries participating in global climate change negotiations?

While existing literature on international negotiation theory is scarce in systematically exploring values within UNFCCC, it still provides insights into countries’ varying climate values and associated bargaining dynamics. Some research emphasizes normative or political-economy principles that shape a country’s position in negotiations (Michaelowa and Michaelowa, 2012b). For example, as Betzold et al. (2012) emphasizes, the principle of common vulnerability made the Alliance of Small Island States (AOSIS) a cohesive and powerful negotiating bloc that overcame bargaining limitations of its individual members. That is, given the shared concern of a looming climate threat, this bargaining bloc adopted a strong negotiation position, helping to advance shared values of fairness and responsibility in addressing climate change among Parties within the UN’s premier climate forum. On the other hand, research suggests that domestic cost–benefit calculations in relation to national climate threats have compelled other countries, such as India and Russia, to reconsider their own values on which they previously based negotiating positions. In these cases, countries shifted from purely defensive and oppositional views toward flexibility and integrative leadership in the global fight against climate change (Michaelowa and Michaelowa, 2012a; Andonova and Alexieva, 2012) – underscoring the influence of shifting values.

Other scholarship similarly focuses on values of justice, fairness, and equity in multilateral climate negotiations. For example, Ringius, et al. (2002) note that the burden-sharing rules that regulate the distributive commitments of emission costs reflect the broader notion of fairness. However, this value’s influence on countries’ climate negotiating behaviors is tempered at times by more narrow, self-serving values. This, in turn, implies that equity and fairness values can conflict with (domestically-oriented) self-interest-oriented values tied to economic, security, or other concerns. Hence, while scholars acknowledge that fairness can be a source of agreement, the question of its predominance is open for debate. To this end, tensions between justice and self-interest-oriented values may translate into a “self-serving bias – where individuals’ judgments of what is fair are often aligned with their own self-interest.” (Brick and Visser, 2015, 80).

These value tensions – both within and between countries – help us understand the imperfections in the actual policy outcomes of multilateral climate talks. Considering fairness and equity, for example, negotiations at COP 13 in 2007 culminated in the establishment of loss and damage mechanisms at COP 19. Similarly, COP 27 in 2022 secured a loss and damage fund agreement to assist developing countries vulnerable to environmental disasters. Such mechanisms are assumed to compensate for the unequal distribution of losses and damages in these countries.^3^ And at COP 16 in 2010, and following multiple years of discussion on the proportionality of harm between the North and South, the notion of historic responsibility was adopted as a way of operationalizing equity (Friman and Hjerpe, 2015). This encoded value accordingly contended that industrialized countries – who are responsible in large part for climate change – should take the lead in mitigation. Yet the primacy of this value began to fade in subsequent COPs. To this end, the 2015 Paris Agreement established a global instrument known as the Nationally Determined Contributions (NDCs), which obligated all countries to outline their mitigation and adaptation commitments every five years to meet collective climate goals. Even so, the NDC framework continues to codify some aspects of fairness and historical responsibility in its (i) differentiation of unequal roles among developed and developing countries and (ii) relaxation of the top-down approach previously seen under the Kyoto regime (Chan, 2015).

In sum, as the studies and evidence reviewed above suggest, values can both support and undermine environmental and climate change cooperation. Indeed, whereas some shared principles – such as shared environmental threat and solidarity – may unify countries’ cooperative positions, others – such as economic opportunities and fear of constraints – may spur fragmentation or deadlock in climate change talks (Costantini, et al., 2016). Thus far, researchers have, by and large, explored a variety of separate principles or values that underlie these competing negotiation pressures. This includes consideration of values such as trust and reciprocity, which may be undermined by self-serving principles and therefore stand to inhibit the climate bargaining process (Schroeder et al., 2025). This also includes the UNFCCC Parties’ prioritization of justice principles as a means of ensuring transparency in climate talks (Albin and Druckman, 2017). Most commonly, however, researchers’ focus centers on the Parties’ adherence to the principles of fairness and, therefore, responsibility (Torstad and Saelen, 2018). And a related commonly considered value is the notion of equity in climate change negotiations, which is interlinked with the actual implementation of common but differentiated responsibilities, as formalized in Article 3 of the UNFCCC (Heyward, 2007).

While these distinct works often emphasize the risk of gridlock arising from divergent preferences (Underdal and Wei, 2015; Stephenson et al., 2019), identifying and considering individual values in UNFCCC negotiations piecemeal is insufficient. To fully understand and foster successful climate change cooperation, one must recover the full spectrum of values underlying this convention, with consideration of how these values shape the conditions among countries. To this end, to the best of our knowledge, only one prior study, conducted by Rosales (2008), attempted to distill and systematize the top-level values surrounding international climate talks (Table 1). To this end, and conducting interviews with various actors, such as policymakers and civil society groups at COPs 6–8,^4^ Rosales (2008, 96) ultimately identified 10 common value groupings “embedded in the discourse of key actors working on emissions trading within the UNFCCC.” While we acknowledge that environmental negotiations are replete with a broad range of values due to the diversity of Parties involved, we find Rosales’ work a notable empirical contribution to the comprehensive and most common understanding of shared value foundations and the differences in values held by various actors operating within the UNFCCC.

**Table 1.** Values framework

Value	Description
Responsibility	Taking account for one’s action, obligation
Power	Social status, prestige, control, dominance, authority, wealth
Fairness	Equity, equal rights, and entitlement; uprightness, decency, justice
Security	Personal safety, stability, national security, social order
Market Logic	Least-cost, efficiency, markets as a value-free arbiter of social goods
Conformity	Restraint of inclinations or impulses likely to upset or violate others, honoring others, amenable, compromising
Universalism	Understanding, appreciation, tolerance, protection of human welfare, broad-mindedness
Self-direction	Independent thought and action, sovereignty, empowerment, freedom
Environmental	Views on the purpose of nature - stewardship, utilitarian, intrinsic
Achievement	Success, accomplishment, personal stimulation, ambition, fulfillment

Taken from Rosales (2008).

In support of this claim, and above and beyond the overlap between Rosales’ values and those discussed further above, we can note more specifically that Rosales includes values of responsibility, fairness, or universalism, which are often identified by scholars as a point of contention between developed and developing countries as it relates to the concept of historic responsibility (Friman and Hjerpe, 2015). Additionally, Rosales’ values of power, security, market logic, and self-direction are commonly discussed by scholars in the context of the domestic determinants of countries’ positions in global climate talks (Joshi, 2015; Andonova and Alexieva, 2012). More direct evidence from individual COPs, as mentioned above, also shows that these distinct values shape negotiations in a more anecdotal sense.

Given the broader overlap in literature and practice with Rosales’ classification of values, we accordingly use the author’s framework in our quantitative analysis to explore (Observer and Party) countries’ values, as expressed in more recent COP High-level Segment (HLS) Speeches over time. We believe that building upon Rosales’ extant framework maximizes the validity of our ensuing measures of values networks at the UNFCCC, since it provides a core set of values that are widely recognized by environmental scholars and practitioners, understanding the bargaining dynamics among the primary negotiators and decision-makers, on which values are commonly considered in prior studies. Importantly, Rosales’ values are also grounded in a rigorous measurement approach. To this end, the author measured values through a series of open-ended interviews administered to 44 key actors in emissions trading across multiple COPs, while engaging with both proponents and opponents of emissions trading (and the UNFCCC) (Rosales, 2008, 94–95). Only those values that were identified by 50% or more of respondents in each such group were retained. Rosales’ 10 corresponding values appear in Table 1. These values encompass the core values outlined in the literature discussed above, including collaborative values related to “Fairness,” “Conformity,” and “Universalism,” as well as more domestically-focused values such as “Security,” “Market Logic,” and “Self-Direction.” In this manner, Rosales’ set of 10 values provides a degree of balance in considering both global collective values and domestic value considerations – consistent with broader recognition of the importance of such balance in prior theoretical and empirical research on global climate change cooperation (Maslin, et al., 2023; Victor, 2011).

In our own approach below, we take a slightly different focus in our UNFCCC units-of-analysis from that of Rosales in concentrating solely on countries giving the HLS Speeches. Using these speeches, we then extract Rosales’ identified values using a keyword-assisted topic model in a manner that also allows for residual themes to be recovered. We believe that this HLS speech-level investigation will contribute to the understanding of bargaining dynamics among the primary negotiators and decision-makers upon which the success of addressing global climate change depends, while complementing prior value-focused insights from scholarship on climate negotiations. Moreover, our approach in extracting 10 overarching environmental values (topics) alongside a comparable number of residual topics from these HLS speeches is also consistent with topic modeling work into values more broadly. For example, Maheshwari et al. (2017, 732) uses topic modeling to classify individual ethical practices on social media into 10 value classes, whereas Borghouts et al. (2023) estimates 10 total topics, five each for very liberal and very conservative tweets, in studying moral values and language usage in COVID-19 discourse. Hence, in further sections, we build on these extant topic modeling approaches to understand countries’ top-level UNFCCC values. However, before we turn to empirically extracting and analyzing these values for our own data, we next provide additional background on the UNFCCC and its negotiation framework.

## 3. UNFCCC negotiation background

Growing concern over climate change in the early 1990s led to the establishment of the UNFCCC, which formally entered into force in 1994. Borrowing from the experience of earlier multilateral environmental treaties (such as the Montreal Protocol), the UNFCCC’s proposed international dialog and structure “bound member states [Parties] to act in the interests of human safety even in the face of scientific uncertainty” (UNFCCC, Nd). These Parties – encompassing the Convention’s formal member nation-states that signed and ratified UNFCCC – have since become the central actors responsible for negotiating climate change agreements under this Framework and globally. Under the UNFCCC, they have done so via the establishment of systematic Party meetings.

Such annual UNFCCC meetings became known as Conferences of the Parties, referred to hereafter as COPs. The first COP took place in 1995 and served to solidify the UNFCCC’s COPs as the supreme formal body for national governments to design multilateral legal tools for reducing greenhouse gases. As such, these COPs function as the central platform uniting diverse national actors with varying concerns surrounding the causes and consequences of global climate change. These conferences have since produced landmark achievements such as the Kyoto Protocol (1997) and the Paris Agreement (2015). Yet, their effectiveness in successfully addressing the climate change problem is subject to debate.

The UNFCCC’s COPs are held annually within two-week windows at rotating locations across the UN’s five recognized regions. To date, there have been 28 annual^5^ meetings between 1995 and 2023. Alongside national government (i.e., Party) participants, these COPs include a number of other actors. This includes Observer States and Observer Organizations, with the latter grouping encompassing groups such as (i) the United Nations System and its Specialized Agencies, (ii) intergovernmental organizations (IGOs), and (iii) non-governmental organizations (NGOs). Notably, Parties are also often self-organized into Party Groupings (e.g., G-77, Arab States, Small Island Developing States, etc.) to facilitate common negotiation positions.^6^

Parties are represented by national delegations “consisting of one or more officials empowered to represent and negotiate on behalf of their government.”^7^ A crucial component to each COP is the delivery of HLS speeches by heads of government or other official dignitaries representing the countries attending each COP as Parties or Observers. These national statements reflect the overarching priorities of UNFCCC Parties and Observers by allowing each national delegation’s high-level representative to outline their country’s positions on climate change issues, express commitments to existing and proposed initiatives, acknowledge progress and obstacles, and/or call for collective action to tackle global climate change challenges. Consequently, these speeches are invaluable sources from which countries’ climate change values can be identified.

As noted above, the UNFCCC’s HLSs are condensed sessions held at each COP that provide countries an opportunity to engage in cooperative climate change dialog on a global scale. In recent years, HLS speeches, on average, range between 500–1000 words and emphasize the most vital climate change priorities for countries in any particular year. The content of these speeches varies thematically. Using a topic modeling approach, Bagozzi (2015) finds that the UNFCCC’s HLS speeches at COPs 16–19 encompass 25 distinct topics and exhibit underlying thematic clusters relating to development, cooperation, the environment, agriculture, and the weather, among others. Lesnikowski et al. (2019) apply a similar topic modeling approach to a larger corpus, finding comparable themes alongside additional themes such as leadership, risk, and vulnerability, as well as those of the Paris Agreement. As such, the diversity of viewpoints expressed in the UNFCCC’s HLS speeches provides important insights into the values and priorities of the UNFCCC’s Party and Observer nations. We accordingly leverage these speeches below to measure countries’ values across recent UNFCCC COPs, which in turn serve as inputs for our social network analysis.

## 4. Text-as-data sample

Our text sample comprises all publicly available HLS speeches delivered during COPs 16–28 (i.e., 2010–2023). HLS speeches were scraped from individual COP webpages dedicated to archiving a particular COP’s content. The specific webpages used are presented in Table 5 of the Supplemental Appendix. Speeches made during earlier COPs are not systematically available on similar webpages and were thus not included in our sample. For the COP 16–28 period, we consider all available HLS speeches made by formal UNFCCC Parties and similar entities such as Observer states (hereafter ‘countries’). The vast majority of these country speeches are given by Parties, given the near-universal country membership in the UNFCCC during our period of analysis. Not every country spoke during each HLS in our sample. Moreover, the relevant COP websites, at times, did not properly archive transcripts for a small number of speaking countries. Altogether, our COPs saw between 71-to-154 countries offer speech texts in any given HLS. The average and median number of country speeches per HLS are 116 and 122, respectively. We treat missing country speeches as missing from our dataset at random during all analyses below. There is no evidence to suggest systematic reasons for missing speeches in our sample, and indeed, at least a subset of our speeches were missing due to archival errors. Reinforcing this contention, past research has shown that missingness in the UNFCCC’s COP HLS speeches is not reliably associated with annual country-level variables such as GDP per capita and CO 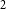 emissions per capita (Bagozzi, 2015).

After scraping all HLS speeches from their associated websites as PDFs, we next extracted and processed the PDFs for automated text analysis. First, we converted all speeches from PDFs to plain text. A majority of our speeches were in PDF format. This allowed us to extract text using standard R routines. A minority of speeches were PDF image files. In these cases, a proprietary optical character recognition (OCR) program, ReadIris, was used to extract speech text, enabling us to assign a speech’s original language for accurate OCR. After converting all speeches to plain text in these manners, we next standardized all speeches to English using Google Translate. A majority of our speeches were already in English. However, the remainder were originally in Spanish, Portuguese, French, Arabic, and – more rarely – other languages such as Russian or Chinese. While Google Translate is sometimes imperfect at the syntactic-level, the bag-of-words framework employed in our topic modeling approach below ensures that these issues are unlikely to affect our analysis. Indeed, this has been shown to generally be the case in the context of bag-of-words models, including topic models (de Vries et al., 2018) as well as for the UNFCCC speeches that we consider here more specifically (Bagozzi, 2015).

Our final sample includes 1,513 speeches in total. These speeches vary in length, with the shortest having only 32 tokens (i.e., words or similar unigrams), the longest having 3,254 tokens, and an average (median) token length of 788 (728). With this sample in hand, we next performed standard preprocessing steps to prepare our speeches for topic modeling. These steps included the removal of standard English stopwords alongside a set of more specialized stopwords for our corpus that helped to address extraneous content such as headers and footers and initial opening remarks.^8^ Alongside this, we removed all numbers, punctuation, symbols, web-links, tokens with fewer than three characters, and sparse terms – while standardizing all retained tokens to lower-case. We then converted all retained tokens to a document-feature matrix (DFM).

## 5. Text analysis

Drawing again upon Rosales, who notes that “[v]alues can be distilled from the discourse surrounding the UNFCCC” (2008, pp. 94), our analysis first endeavors to recover HLS-speech-level (and hence country-year-level) estimates of the 10 values discussed earlier. This is achieved by applying a keyword-assisted topic model (KeyATM) to the speech data described above. Following this, we use our KeyATM-derived values-based measures to construct and analyze a series of network measures in relation to the changing value networks underlying COPs 16–28.

### 5.1 Keyword assisted topic model

As mentioned above, our first analysis step applies a specific topic model known as a KeyATM (Eshima et al., 2024) to our speeches in order to recover measures of each speech’s conveyed values, as based upon the values presented above in Table 1. We selected the KeyATM for its (guided) flexibility in allowing us to recover and, hence, measure values for our speech texts. Given the large number of climate change values we wish to capture, as well as the specialized nature of both these values and the HLS speech texts we consider, a KeyATM approach was deemed more optimal than developing and applying sentiment-like dictionary methods to our speeches.

To this end, the KeyATM allows us to identify groupings of words associated with latent topics based on word co-occurrences across our speeches. From these topic-specific word groupings, we can then measure the topical proportions underlying each associated speech in relation to each of our value-specific topics. Two key strengths of the KeyATM in this regard are (i) its allowance for researchers to specify keywords (and hence topic labels) in relation to a chosen number of topics prior to the estimation of one’s topic model and (ii) its flexibility in estimating a second separate set of non-keywords topics alongside its estimated keywords topics. For keyword-assisted topics, keywords are incorporated through a Bernoulli-governed mixture of two categorical data-generating processes (d.g.p.’s). The first reflects draws of topic-words from that topic’s set of user-assigned keywords, whereas the second draws topic-words from that topic’s standard topic-word distribution^9^ (Eshima et al., 2024, 732). During estimation, this allows the KeyATM to flexibly place greater importance on a keyword topic’s specified keywords by increasing the prior means and variance of these assigned keywords relative to non-keywords – while still allowing one’s documents and their observed word rates to inform this process (Eshima et al., 2024, 733).

In these manners, we are able to assign distinct value-specific keywords to a subset of our topics during topic estimation while also separately estimating a subset of non-value topics. This, in turn, allows us to recover topics specific to each of our 10 values of interest in a manner that separately partitions any distinctly non-value-specific content (as non-keyword topics) from these value topics. Using the model’s (keyword) topic estimates, we can then obtain subsequent measures of each topic’s prevalence within our speech texts for use in our subsequent network analyses. While the above components serve as our primary justification for choosing a KeyATM, we can also note that – relative to common topic modeling alternatives for such tasks – the KeyATM also has the advantages for exhibiting (i) better document classification performance, (ii) more readily interpretable results, and (iii) reduced sensitivity to one’s choice of topic number (Eshima et al., 2024). To this end, we can note, for example, that because the KeyATM utilizes keywords to constrain topic-word distributions rather than relying solely on word co-occurrences, stemming terms is no longer as necessary for the estimation of semantically meaningful topics. This allows us to follow past KeyATM research (e.g., Eshima et al., 2024; Diaf, 2022; Eisele and Hajdinjak, 2023a) in leaving our keywords and topic results un-stemmed so as to maximize interpretability while minimizing the blurring of semantically similar terms.

For our KeyATM application, there are several quantities that we must define a priori as researchers. One such quantity encompasses the sets of keywords to be used in recovering each of our value topics. We ultimately assigned 5–7 keywords for each intended value topic. These selected keywords appear in Table 2. To identify these keywords, we first developed a list of approximately 10 candidate keywords from the original illustrative terms for each of our ten values as presented in Rosales (2008) and Table 1, as well as from the corresponding discussion of these values in Rosales (2008). While this initial set of keywords offered several promising terms, many associated words were too academic in focus as opposed to the types of terms used in regular HLS speeches. Thus, two coauthors of this paper qualitatively and independently read all 1,513 speeches in our corpus to identify other candidate keywords. This expanded our candidate keyword list to between 34–87 candidate keywords per value. A final set of 5–7 keywords was then chosen from each list based upon (i) discussions among both qualitative readers and (ii) a subsequent review of a subset of these keywords’ actual frequencies across our speeches. A keyword frequency plot appears in Figure 8 of the Supplemental Appendix for our final selected keywords. There, one can note that a majority of our keywords fall above the 0.1% corpus threshold advised by Eshima, et al. (2023) for the KeyATM.

**Table 2.** Selected keywords for keyATM

Value	Keyword 1	Keyword 2	Keyword 3	Keyword 4	Keyword 5	Keyword 6	Keyword 7
Responsibility	Need	Implementation	Action	Efforts	Address		
Power	developing	developed	capacity	power	strong		
Fairness	balanced	differentiated	fair	kyoto	protocol		
Security	vulnerable	loss	people	impacts	threat	floods	damage
Market-logic	economic	resources	development	economy	finance		
Conformity	commitment	agreement	cooperation	community	support		
Universalism	generations	countries	world	international	future		
Self-direction	country	national	domestic	citizens	republic		
Environmental	environment	environmental	green	greenhouse	nature		
Achievement	ambitious	progress	goal	achieve	success		

*Note:* Five keywords are used for all topics except for the Security topic, which uses seven keywords.

Having defined our keywords based on the above approach, we proceeded to assign several remaining quantities for KeyATM estimation. Alongside the 10 keyword-assisted topics outlined above, KeyATM users also must assign the number of non-keyword topics to be estimated. Given that (i) the thematic content within our corpus is unlikely to only correspond to values and (ii) past research has found upwards of 20–25 topics to underlie these HLS speeches (Bagozzi, 2015; Lesnikowski et al., 2019), we specify our KeyATM to recover an additional 10 topics beyond our 10 value topics. This relatively large number of auxiliary topics allows us to best partition the wide-rage of “residual” (e.g., region- and COP-specific) content from the value-oriented content contained in our speeches. At the same time, our own exploration of our KeyATM’s non-value topics’ content and (relatively low) document-level proportions suggests that these topics are sparse and, at times, less coherent than our value topics. This implies that increasing our non-keyword topics beyond 10 is unlikely to recover additional meaningful topical content^10^ Finally, we set the number of iterations to our KeyATM at 5,000 to ensure convergence. We present model fit diagnostics that affirm our choices of iterations and topic number in Figure 9 (Supplemental Appendix).

**Figure 1.**
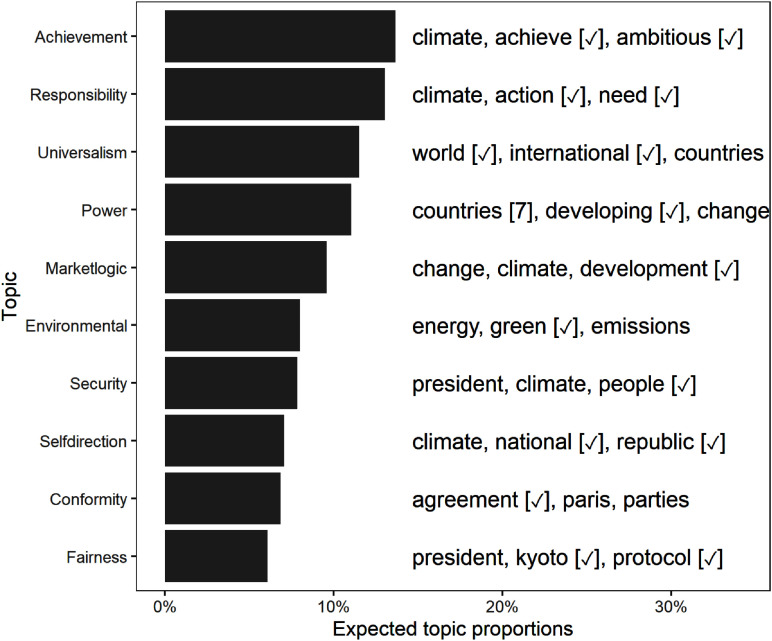
Keyword-based topic prevalence across HLS speech corpus. The top three words associated with each topic appear to the right of each topic. Checkmarks denote keywords assigned to a topic by the authors during KeyATM estimation.

Results from our KeyATM can be found in Figure 1. This figure presents the relative frequency of our 10 estimated values topics across our corpus alongside each topic’s top three tokens^11^ A table displaying the top 10 tokens for each of our 10 values topics and a separate table presenting the top 10 tokens for our 10 non-keyword topics appear in the Supplemental Appendix. The Supplemental Appendix also presents qualitative interpretations of each topic, based on a review of the top tokens for each topic and the most highly associated speeches. Altogether, our KeyATM does an intuitive and coherent job of recovering our 10 value topics, whereas the non-value topics do not appear to reflect distinct value-specific content. As can be seen in Figure 1, the most common topics in our corpus are “Achievement”, “Responsibility”, and “Universalism”, whereas the least common topics correspond to “Self-direction”, “Conformity”, and “Fairness”. That being said, we do not see extensive variation in expected topic proportions across our 10 value topics. Figure 1 also demonstrates – via its 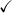 symbols – that all 10 values topics see at least one of our assigned keywords for that topic within their top three tokens. While KeyATM estimation places more weight on these assigned keywords by design, deficiencies or inconsistencies in the appearance of such keywords in topword results do arise in KeyATM applications – which is at least partly treated as evidence for the model’s failure to identify meaningful terms (Eshima et al., 2024, 743) or relative under-performance in coherent topic recovery (Eisele and Hajdinjak, 2023b, 1033). Hence, our finding that each value topic retains at least some assigned keywords within its topwords vector suggests that our model is coherently and distinctly recovering all 10 value topics.

The above observation and the additional evaluations that we do of our KeyATM in the Supplemental Appendix together suggest that our keywords and values topics are indeed recovering distinct and value-consistent constructs. To this end, the qualitative readings of our keyword-based topics help to confirm that the HLS speeches and top tokens identified for our ten value topics align closely with our expectations concerning each value outlined in Table 1. These qualitative assessments also add further nuance to our understanding of how certain values are discussed at the UNFCCC. For example, the speeches associated with our “Conformity” topic were found to be more aspirational and forward-looking than we initially anticipated. These are two qualities that make sense when we consider this topic’s periods of highest influence in the network analysis further below. Likewise, our “Environmental” topic focused more on addressing the global climate change problem^12^ than on climate change’s broader environmental consequences. This is consistent with the overall focus of HLS speeches, in the sense that aside from discussions of “Security ”- oriented consequences, relatively less attention is systematically paid to climate change’s broader environmental consequences in relation to aspirations of or achievements in climate change mitigation.

In addition to validating our 10 value topics in the manner discussed above, we also qualitatively review our 10 non-keyword topics in the Supplemental Appendix. We find in most cases that the associated topics are less coherent and more region- or COP-specific than our 10 value topics. For example, our non-keyword topics appear to capture fairly unique concerns held by small island states, broader Latin American regional concerns, territorial disputes, COP 28-specific discourse, or the implications of climate change for mountains and the Arctic. This implies that our non-keyword topics encompass content that is largely distinct from the values discussed earlier. Accordingly, we view our auxiliary topics as performing as expected in capturing “error term”-like content within our COP HLS speeches that does not closely align with our 10 value topics. Tables 9–12 of the Supplemental Appendix also further demonstrate the robustness of our value topic interpretations to changes in the number of non-keyword topics estimated.

Using our KeyATM’s 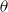 parameter – which corresponds to the model’s speech-topic distribution – we then recover topic proportions for each HLS speech in our corpus. This provides us with a proportion-based measure of the share of each annual speech that can be attributed to each of our 10 value-based topics. For our speeches, these value topic-specific proportions range from 0.0001–0.6533, with each topic’s average proportion across all speeches ranging from 0.0609 – 0.1367. Next, we utilize these recovered proportions to construct and analyze the social networks outlined below.

## 6. Network methods

Social Network Analysis and Network Science have a long history of being applied to political and organizational networks, and we will build on this literature (Knoke et al., 2021; Koskinen and Edling, 2012; Lazer, 2011; Neal, 2014). In this section, we first review the weighted bipartite networks (Wasserman and Faust, 1994) we constructed from the HLS speeches; then, we review basic network descriptive statistics and influence measures. Last, we examine how countries and values cluster across space and time.

### 6.1 Weighted bipartite networks

In this work, we model each COP network as a weighted bipartite graph 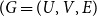 , where 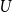 denotes HLS-participating countries, 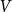 denotes values, and 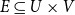 represents weighted ties between them. A graph 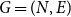 consists of nodes (vertices) 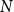 and edges 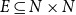 linking pairs of nodes. Networks can equivalently be represented by an adjacency matrix 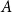 , where 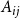 encodes the relation 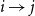 . For unipartite networks ( 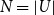 ), 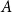 captures within-set relations (e.g., alliances among countries). In bipartite networks, edges occur only across sets ( 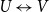 ), prohibiting within-set ties. One can obtain one-mode projections via 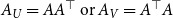 , representing country–country or value–value relationships, respectively. See Neal et al. (2024) for a review of bipartite networks and their projections.

**Figure 2.**
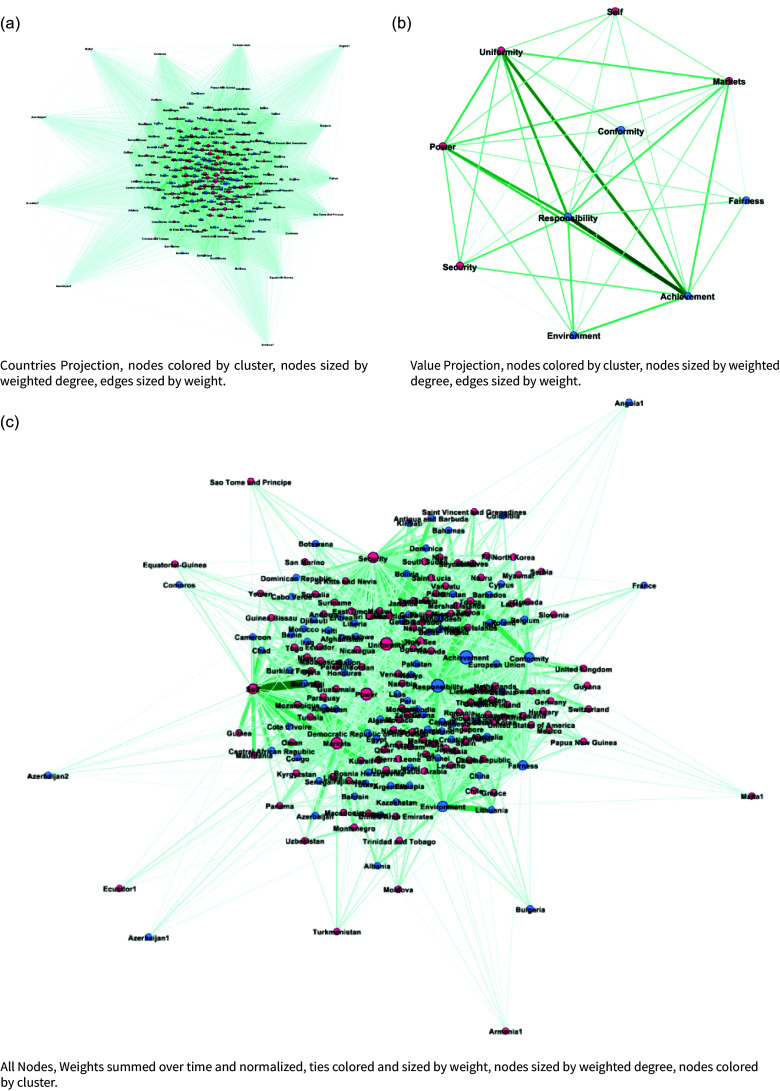
Network plots of (a) country-to-country projection, (b) value-to-value projection, and (c) Bipartite country-to-value network. All nodes are scaled by degree, and edges are scaled by weight. Node positioning derived from ForceAtlas2, a fast, continuous, force-directed layout algorithm designed for visualizing small to large networks (Jacomy et al., 2014).

Here, the two node sets are countries and values, and the edges represent predicted weights between country pairs (in this case, we use the outcome of the KeyATM prediction). We refer to these as *political value networks* (or simply *value networks*). Figure 2 visualizes these networks with tie weights summed over the 13 annual observations. We construct them by projecting the bipartite weighted country–value network (by topic and year) to obtain COP value networks for 2011–2023.^13^ The KeyATM naturally generates weights for the relationship between a country and value (e.g., the US and Achievement, Environment, and Responsibility). An edge exists between a country and a value if the estimated weights exceed zero.^14^

### 6.2 Network descriptive

We focus on three core graph metrics, which help us understand properties of these networks: (1) measures of connectedness, such as density and degree; (2) centrality measures and other measures of influence and/or power; and (3) clustering metrics. We start with one of the most fundamental measures, which is known as graph *density*, the ratio of observed to possible edges, interpretable as the probability of a tie in a random graph (Butts, 2006). In this same set of metrics, we look at the *weighted degree distribution*, which describes how the total connection strength (sum of edge weights) varies across nodes in the network (Wasserman and Faust, 1994). For example, in a unipartite social network, it reflects how many (or how strongly) individuals connect to others; and in a bipartite network, such as one linking people to organizations, it captures how many affiliations each person or organization has, weighted by interaction strength. The next set of key metrics centers around node-level characteristics. Node-level influence is captured by *centrality* measures (Freeman, 2002), including: degree (number of incident edges), betweenness (share of shortest paths passing through a node), closeness (inverse distance to all others, reflecting potential information flow), and eigenvector centrality (influence weighted by neighbors’ centrality) (Wasserman and Faust, 1994; Freeman, 2002). Finally, we examine *community detection*, which identifies node groups more densely connected internally than externally (Harenberg et al., 2014). Communities (or clusters/modules) reveal latent structure, reduce network complexity, and highlight functional or social roles (e.g., friend groups, biological modules, alliance clusters).

### 6.3 Network clustering

There is a robust literature in community detection centered around different metrics such as generalized eigenvector centrality or tensor co-clustering (Almquist et al., 2024), betweenness (Chang et al., 2015), or modularity (Newman, 2006). However, the literature on community detection for bipartite networks is less developed (Alzahrani and Horadam, 2015; Wu et al., 2024). In this work, we focus on Friel et al.’s (2016) approach to bipartite modeling dynamic networks in a latent space as a means to generate bipartite clusters. In their work on the interlocking nature of corporate board members, Friel et al. (2016) characterize their bipartite network of boards and members as a Bayesian hierarchical model, enabling them to assess the latent space of board members through Markov Chain Monte Carlo sampling. Similarly, we apply this method to our own data, providing the sampler with our annual country-value networks, randomized initial locations, and suggested initial parameters. As the sampler converges with additional iterations, we take the final sample and use each node’s latent-space coordinates to plot (Figure 7) and group nodes based on their similar tie structures using a k-means clustering algorithm described by Hartigan and Wong (1979).

To determine the optimal number of clusters, we employ a multi-metric validation approach. We assess cluster quality using three complementary methods: Bayesian Information Criterion (BIC) from Gaussian mixture models (Fraley and Raftery, 2002), the Gap statistic (Tibshirani et al., 2001), and average Silhouette width (Rousseeuw, 1987). Figures 6a, 6b, and 6c show the BIC, Silhouette, and Gap statistic results, respectively, across 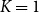 to 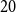 clusters. All three metrics converge on a low-clustering solution, with the BIC and Gap statistic selecting 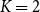 and the Silhouette selecting 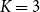 . Using a simple majority vote across metrics, we select 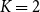 as the final number of clusters. The results show disparate nations joined together based on the content of their speeches. This method allows us to produce both clusters within the country and values representative of their tie structures across time, which we then use as node attributes in our later modeling to reflect the relationship between topics and nations over time (Figure 7).

## 7. Network analysis results

### 7.1 Descriptive statistics

**Figure 3.**
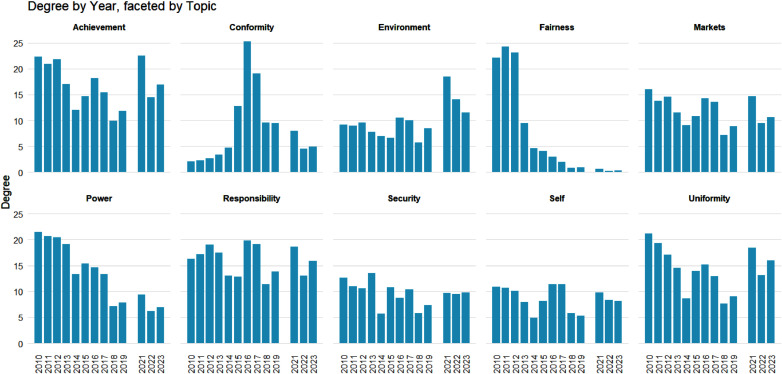
Degree by year by topic for all 13 years. The degree is calculated from the weighted graphs that include forcing the sum of the standardized topic proportions to one for a country-year observation in case a country-year was missing a topic.

We begin our network analysis with a visual of the bipartite network and its projection in Figure 2. This figure consists of the summed weighted ties between countries and values over the 13 annual observations. We can see a tight cluster of countries and the central importance of “Responsibility” and “Power” in the values network. We find that the mean degree over all time periods ranges from 0.63 to 1.37 and the density ranges from 0.031 to 0.068, where degrees are normalized to sum to 1 within each country; see Table 3 for the normalized statistics, calculated across the annual networks. Overall, we see more connectedness and activity in the middle of the sample period, with a general decline in connectedness in the last few years. Many of the later ties are captured by achievement and uniformity, and there is a slight decline in the number of countries giving speeches. Finally, we examine the degree distribution of the values (Figure 3) over 13 years. Here we see that equity-adjacent values such as “Uniformity,” “Fairness,” and “Conformity,” appear as standouts early on before giving way to issue-specific values such as “Security,” “Power,” and “Environment,” and then later to a focus on “Achievement” and “Conformity.” As elaborated upon in the Discussion section, these patterns likely reflect value shifts away from (debates over) post-Kyoto Protocol-era priorities to the lead-up to and then implementation of the Paris Agreement.

**Table 3.** Mean degree and density over 2010 to 2023. All degree terms are normalized within the country (i.e., they sum to one)

Statistic	Mean Degree (normalized)	Density (normalized)	Mean Degree	Density
Min	0.6667	0.0314	0.6348	0.0314
Max	1.4460	0.0682	1.3799	0.0682
Median	1.1455	0.0540	1.0916	0.0540
Average	1.0928	0.0506	1.0348	0.0515
SE	0.2655	0.0132	0.2579	0.0125

**Figure 4.**
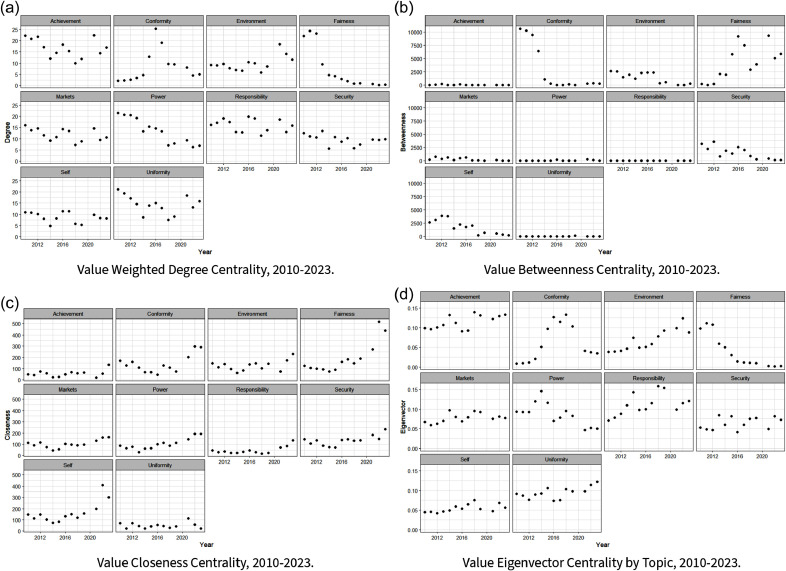
Centrality scores by value for all 13 years of HLS data.

#### 7.1.1 Centrality measures

Next, we examine how our network centrality measures (Wasserman and Faust, 1994) shift over time. This offers unique insights into the changing position of values within our network – a quality that both elucidates core value shifts over time and the top countries associated with these values. To this end, we first look year over year at the centrality of each value topic (see Figure 4), where we can see temporal trends between different centrality measures and values. Certain values, such as “Conformity,” have temporal volatility across centrality scores, while others, like “Achievement,” vary between but not necessarily within a given centrality score. In at least two of our four centrality cases, we observe initial focus on the topics of “Fairness” and “Power” that decrease in each case over time, while other topics like “Responsibility” and “Security” ebb and flow. According to Eigenvector, degree centrality, and to a lesser extent, degree centrality, the “Environment” topic experienced an increase in importance after 2020. “Achievement” has likewise seen modest, very recent gains across at least three measures. Still, no topic has exhibited over time shifts quite like “Fairness” albeit in different directions depending on the centrality measure considered.

Here, we consider eigenvector centrality more explicitly. In this case, a country has high eigenvector centrality if it is connected to other highly central countries. This allows us to interpret high eigenvector centrality as a measure of connectedness over the most popular values in our network. In Table 4, we see that the European Union and closely affiliated European countries (specifically, Belgium, Ireland, Romania, and Austria) dominate and typically exhibit “Conformity,” “Responsibility,” and “Achievement” as the most central values – albeit with some attention to “Power” and “Fairness” early on in our period of analysis. Secondarily, China and several North African developing countries can also be observed during this early period in Table 4, with central values again centered on “Power” and “Fairness.”

**Table 4.** Maximum eigenvector centrality by year with top country or value selected

Year	Country	(max) Eigenvector Centrality	Value	(max) Eigenvector Centrality
2010	Austria	0.09542331	Fairness	0.09913991
2011	South Sudan	0.09741075	Fairness	0.11266134
2012	Algeria	0.10127439	Fairness	0.10994726
2013	Eritrea	0.10238597	Power	0.11969413
2014	China	0.12760862	Power	0.14801889
2015	European Union	0.10889562	Power	0.11853160
2016	Romania	0.10072923	Conformity	0.12751230
2017	Thailand	0.10148495	Conformity	0.11746941
2018	European Union	0.13856917	Responsibility	0.15960340
2019	European Union	0.12796338	Responsibility	0.15500270
2021	Ireland	0.10654825	Achievement	0.12301909
2022	Belgium	0.12337874	Achievement	0.12950998
2023	European Union	0.11903433	Achievement	0.13520630

**Figure 5.**
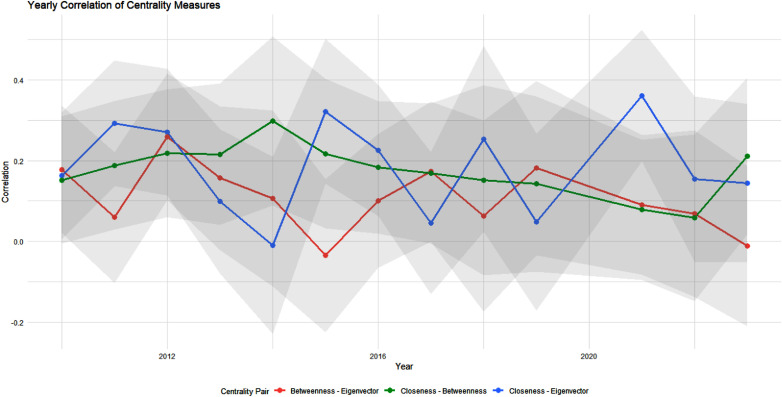
Correlation between centrality measures over time. Pairwise average correlation: eigenvector centrality with closeness is 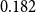 , eigenvector centrality with betweenness is 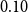 , and closeness centrality with betweenness is 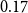 .

All of the centrality measures are positively correlated, with average pairwise correlations of 0.18 between eigenvector and closeness centrality, 0.10 between eigenvector and betweenness centrality, and 0.17 between closeness and betweenness centrality. This, however, varies over time (see Figure 5). We see a maximum correlation of 0.361 and a minimum correlation of −0.03. This correlation among these measures is expected to be largely positive. However, we do observe a few years of negative pairwise correlations between centrality measures. Still, most years and measures are positively correlated, but always under 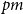 0.4 (this is typically viewed as weak to moderate correlation Taylor, 1997). As shown in prior work (Wasserman and Faust, 1994), different centrality measures in social networks tend to be highly correlated with one another, as shown in Figure 5 for our case. This overlap reflects the fact that many centrality metrics capture similar underlying dimensions of node influence or connectivity within the network. Countries that are highly central due to being closely embedded (closeness) are not always those that exert influence through bridging roles (betweenness) or through connections to highly connected ideas (eigenvector centrality). This highlights that agenda-setting power, discursive brokerage, and thematic embeddedness represent overlapping yet non-interchangeable forms of influence in climate negotiations.

While we withhold full interpretation of these results for our Discussion section, we can briefly note at this juncture that a review of the annual speeches associated with our identified “top” countries in Table 4 suggests that their identification was primarily based on their exclusivity of focus on a particular value in a particular year. For example, Eritrea’s 2013 speech was primarily focused on power imbalances between developed and developing countries, especially surrounding these groupings’ disparities in willingness and urgency to combat climate change – directly in line with our “Power” value topic. Likewise, Thailand’s speech in 2017 centered on the need for collective commitments and efforts in researching collective outcomes concerning the Paris Agreement and more generally – closely aligning with our value topic of “Conformity.”

For the values themselves in Table 4, we see that there is a shift from “Fairness” to “Power,” to “Conformity,” to “Responsibility,” and finally to a focus on “Achievement.” Last, we find a high correlation between OECD countries and eigenvector centrality of 0.16 (with SE of 0.03 and statistically significant at 0.05 alpha-level), compared to – for example – African nations which correlate to eigenvector centrality is −0.19 (with an SE of around 0.03 and statistically significant at 0.05 alpha-level). Together, this demonstrates the core qualities of OECD countries compared to developing countries, such as those in Africa, which are the second most common geographic group in Table 4.

### 7.2 Clustering analysis

**Figure 6.**
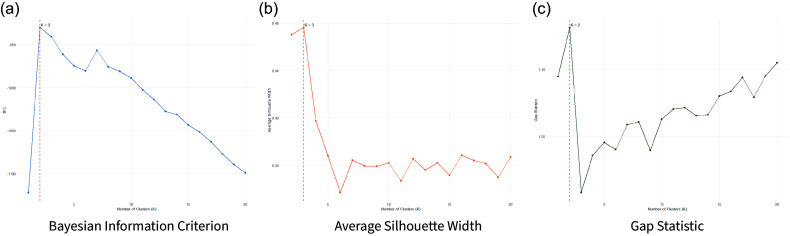
Comparison of clustering validation metrics across cluster solutions.

**Figure 7.**
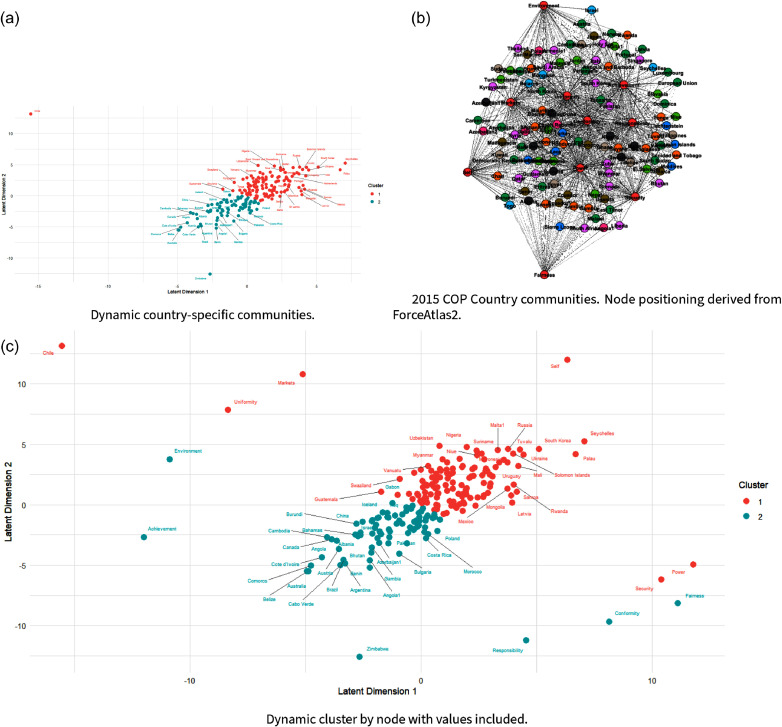
Colors by k-means cluster, all nodes and ties included.

**Figure 9.**
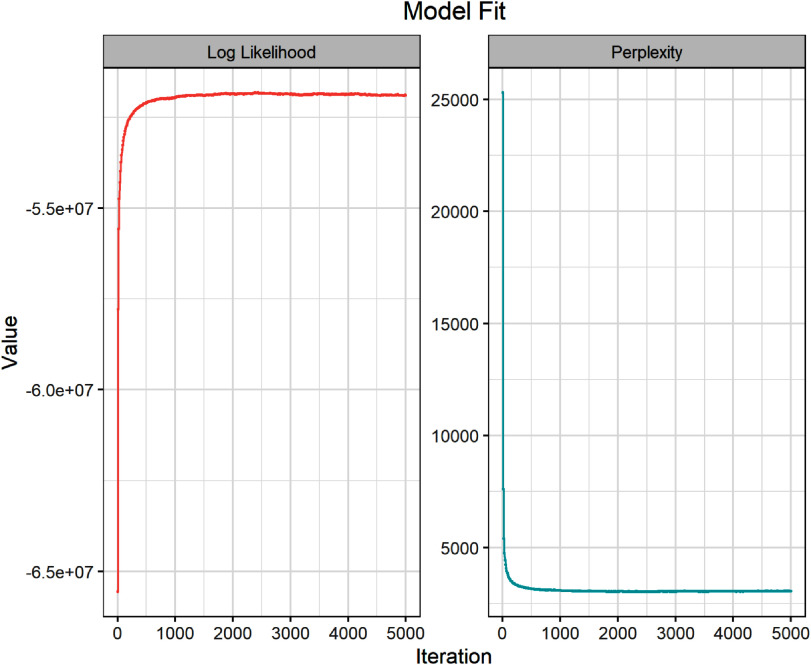
Model fit diagnostics for KeyATM. The left-hand subplot presents within the model’s sample log-likelihood, whereas the right-hand subplot presents the model’s predictive log-likelihood (i.e., perplexity). Higher values of within-sample log-likelihood are preferred, whereas lower values of perplexity are preferred in implying more model confidence and accuracy in predicting unseen text-as-data. Increasing stability in values obtained for each quantity as the number of model iterations (x-axis) increases is, in turn, a sign of model convergence.

Using Friel et al.’s (2016) characterization of the bipartite network as a Bayesian hierarchical model through the application of Markov Chain Monte Carlo sampling, we analyze the annual nation-topic networks using the IrishDirectorates package in R (Rastelli, 2024). To find final clusters, we use the k-means algorithm from Hartigan and Wong (1979). The results can be seen in Figure 7. We find two core groups across countries and values using three fit metrics: The Bayesian Information Criterion (BIC) evaluates model fit while penalizing complexity (inverted from standard lowest best) (Schwarz, 1978), the Average Silhouette Width measures within-cluster cohesion and between-cluster separation (Rousseeuw, 1987), and the Gap Statistic compares within-cluster dispersion to that expected under a null reference distribution (Tibshirani et al., 2001) (see Figure 6).

We find at least some evidence of clustering among developed countries and developing countries. As with centrality scores, we see that developed countries are more central than developing countries. This is consistent with scholarship on UNFCCC negotiations, which has extensively found greater influence among developed countries than among developing countries in this venue (Falzon, 2021, 185). That being said, our dynamic clusters by nodes and values tend to place major emitters such as the United States, China, and Russia/The European Union in distinct but intuitive value clusters intersecting with “Self Direction,” “Responsibility” and “Fairness”/“Achievement,” respectively. This underscores the challenges of reaching consensus in UNFCCC negotiations, to the extent that these key emissions players appear relatively distinct in their core negotiation intentions.

## 8. Discussion

On the whole, our combined network and text analysis illustrates that the top-level values held by negotiating countries at the UNFCCC have evolved over recent COPs, with a subset of developed countries serving as likely drivers. At the same time, this analysis also suggests that many of the most influential countries and informal bargaining blocs at the UNFCCC tend to emphasize distinct top-level values, and that a diverse range of top-level values remains active in this forum to this day. Underscoring these points, we see strong evidence of temporal change of the most central value topics (Table 4), with certain values being more volatile in their importance over time (Figure 4). Our networks accordingly show varying activity over the 10 years we consider, illustrating how different countries and values change year to year. Looking across our values, we can further note that our bipartite network project identifies a central importance of “Responsibility” and “Power” in its values projection – suggesting that values associated with the distribution of power in the international system and historical climate change responsibility stand out in underpinning all of the top-level values identified in this negotiation period.^15^ And, as alluded to above, it furthermore appears that developed countries, and in particular the European Union and European nations, are central to shaping much of the HLS’s (evolving) values,^16^ with large scale transition from discourse over “Fairness” to an emphasis on the actual attainment of climate goals and ambitions (“Achievement”).

As evident from these aggregate findings and our specific network results, there is at least some year-to-year persistence in our most influential UNFCCC values during the post-2010 period. This can be seen, for example, in the two-to-three-year persistence of top values identified in Table 4. However, there is also clear evidence of turnover in these values across our entire sample period. Between 2010 and 2012, “Fairness” substantially influenced discourse within the UNFCCC’s climate talks. This is most clearly demonstrated in Figures 4a, 4d and Table 4, and secondarily in Figure 3. The prominence of “Fairness” as a value during 2010–2012 can be related to several outcomes during this period at the relevant COPs. In 2010, at COP 16 in Cancun, after prior years of fragmentation and stalemate in negotiations,^17^ the UNFCCC’s Parties made significant progress towards comprehensive agreements with an emphasis on helping developing nations (Liu, 2011). This emphasis, in keeping with lingering discourse over the Kyoto Protocol during this period, was predicated upon the need to compensate for the historically unfair distribution of CO 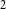 emissions and inequality in individual development patterns.

In particular, COP 16 saw the conclusion of a series of Cancun Agreements^18^ that established the Green Climate Fund (GCF). The latter GCF was directed towards providing financial support for climate change initiatives in developing countries (Antimiani et al., 2017) – thus speaking directly to historical issues of fairness in climate change contributions and resources. To this end, the GCF specified capacity-building and resilience mechanisms for vulnerable developing countries via technology transfer and the Fast-start Finance initiative, among other innovations. At COPs 17 and 18, these and other initiatives were welcomed and further developed. In Durban, the governing instrument for GCF was designed, the goal of mobilizing funds by 2020 to help developing countries was outlined, and the Technology Mechanism became fully operational (Tanunchaiwatana, 2012). These steps became fundamental for addressing inequality and involving low-income countries in collective negotiation over establishing a legally binding universal agreement by 2015 as specified by the Durban Platform (Rajamani, 2012). In Doha, developed countries advanced their commitments to support developing nations and set pathways toward formalization of the loss and damage mechanisms for vulnerable states (McNamara, 2014). The latter was guided by fairness principles, including equitable access to financial support, recognition of justice, and the right to redress.^19^

By 2013, as underscored in Figures 3, 4, and Table 4, the UNFCCC’s core values started to gradually shift. While COP 19 in Warsaw^20^ formalized loss and damage mechanisms on “Fairness” grounds, the goal of a universal agreement binding nations to emissions reductions took center stage (European Parliament, 2013; United Nations Environment Programme, 2013). This likely contributed to a decrease in “Fairness” as the dominant value within UNFCCC climate discourse, instead shifting discussions to “Power,” wherein participating countries likely saw increased disagreement over power dynamics in future agreement structures. This trend is evident in Table 4, and secondarily Figure 4c and Figure 3, with the rise in the “Power” value in 2013–2025. And in line with these observations, evidence suggests that power imbalances did indeed lead to a battle between developing and developed countries about the nature of the future agreement – be it top-down (prescriptive), bottom-up (facilitative), or hybrid (Rajamani, 2014). In addition, negotiations in Warsaw set preliminary requirements for national action plans under the NDCs framework, which further divided UNFCCC Parties while raising the salience of other values over that of “Fairness.”

To this end, NDC formulation underscored capacity gaps and power inequities among developing and developed countries (UNFCC, 2023). Indeed, despite developed countries being urged to assist developing nations, evidence suggests that developed countries’ (in)ability to mobilize finance and technology in support of NDCs may foster asymmetry and tensions between developed and developing countries (Climate-Strategies, 2022). In Lima at COP 20, the debate over shortfalls in efforts by large emitters contributed to tensions between developed and developing nations. The Lima Accord – a non-binding treaty that advances NDCs – emerged as a pressure point in which least developed and vulnerable countries were pushed by industrialized countries to reduce their emissions, thus putting the responsibilities of each group of countries on a more equal footing (Leber, 2014). This again highlights the power imbalance among states during this period, wherein less powerful developing countries sought to emphasize fairness and differentiation of obligations while stronger countries refused to accept such objections.^21^

Yet another major turning point for the UNFCCC’s climate talks occurred in Paris in 2015. The culmination of COP 21 saw countries overcome many of the imbalances and disagreements outlined above to reach a landmark breakthrough in the Paris Agreement. In response, “Conformity” came to the fore, underscoring solidarity between the UNFCCC’s Parties in both the establishment of the Agreement and in looking ahead toward its implementation. To this end, and for the first time in UNFCCC negotiations, the Paris Agreement brought “all nations into a common cause to undertake ambitious efforts to combat climate change and adapt to its effects, with enhanced support to assist developing countries to do so.”^22^ It further codified preceding actions and mechanisms developed at earlier COPs. It reaffirmed the “obligations of developed countries to support the efforts of developing country Parties” in building a sustainable future.^23^ This resolved a number of “Fairness”- oriented and Power ”- based disagreements concerning mitigation responsibilities between the developed and developing countries. It further promised a collective approach to addressing global challenges. At the time, the Paris Agreement was perceived as a new chapter in climate negotiations wherein global climate efforts were tied together and where consensus, cooperation, and common goals subsumed past frictions (Dimitrov, 2016). This naturally led to a greater (aspirational) focus on “Conformity” over this agreement and future climate cooperation among participating countries – as depicted most explicitly in Figures 4a–4d and Table 4.

During the three COPs following Paris, this collective and often aspirational spirit was reinforced. Much of this continued focus on “Conformity” was predicated upon country efforts to implement the Paris Agreement. For instance, the 22nd COP session in Marrakesh in 2016 set the priority 2018 deadline “for completing the nuts-and-bolts decisions needed to implement the agreement fully.”^24^ Remarkable progress was noticed during this period within negotiations, given the focus of Parties to undertake efforts to ratify and implement the provisions of the Paris Agreement. COP 23 in Bonn reiterated the Parties’ commitments to collective action, facilitated dialog between developed and developing countries, and advanced efforts to define the Paris Rulebook, which was further discussed in Katowice the following year. COP 24 then formalized most of the technical discussions surrounding the Paris Agreement and promised to be a guiding light for actionable climate talks (UNFCCC, 2018).

With the general excitement surrounding the Paris Agreement subsiding by 2019, awareness of the “Responsibility”-dimensions of the agreement became more apparent – as reflected in Table 4 and to a degree in Figure 3. Being held in Madrid a year prior to a global pandemic, COP 25 saw Parties fail to agree on Article 6 of the Paris Agreement relating to cooperative approaches (Obergassel et al., 2020). This and other trends of stagnation and backsliding vis-à-vis the Paris Agreement likely led to less enthusiasm for “Conformity”, and a renewed debate over nations’ “Responsibility” in the climate change arena. The latter trends were perhaps further reinforced by other efforts at this COP, including nations’ achievements in strengthening the “implementation of the Warsaw International Mechanism for Loss and Damage” and “adopted an enhanced gender action plan” (Erbach, 2020). More generally, discussions over the responsibilities and technicalities of the Paris Agreement’s implementation arguably diverted negotiation attention away from the primary issue of the threats posed by climate change. This led to somewhat disappointing outcomes at COP 25. Ultimately, these trends were best captured in Sengupta’s account of COP 25, which was argued to have demonstrated “how disconnected many national leaders [were] from the urgency of the science and the demands of their citizens” (Sengupta, 2019).

After a COP postponement due to the global COVID pandemic, countries again reconvened in 2021 in Glasgow at the 26th session. Given the intensification of extreme weather events and slow progress on addressing climate change problems at earlier COPs, “Environment” values and environmental priorities seemingly took due attention in 2021 (Tobin and Barritt, 2021) and thereafter. This trend in the importance of our “Environment” topic can be clearly seen in Figures 4a, 4c, and 4d. At the same time, progress in implementing the Paris Agreement also understandably appeared to take hold via increased attention to “Achievement” – as is especially evident in Table 4 and to a certain extent in Figure 3. Qualitative evidence also supports these patterns. At COP 26, participating countries signed the Glasgow Climate Pact and eventually agreed on the Paris Rulebook.^25^ Alongside this, global commitments relating to – and associated progress on – fossil fuels, forests, and vehicle emissions were made. At COP 27 in Sharm El Sheikh, Parties further agreed on loss and damage funding, mobilized more support for developing nations to address the severity of the environmental anomalies, and expressed the intention to hold businesses and institutions accountable.^26^ Finally, 2023’s COP 28 in Dubai drew more attention toward renewables, signaled a willingness to end the fossil fuel era, and ramped up practical climate solutions such as the scaling up of finances to expedite efforts to combat climate change.^27^ Hence, recent years have seen an increased and sustained focus on post-Pairs achievements on, and concerns over, (i) the environmental threat of climate change and (ii) renewable energy as a core solution, thereby leading to an increase in the importance of associated “Achievement” and “Environment” values.

## 9. Conclusion

This paper extracts and analyzes value networks for the 13 most recent Conferences of the Parties (COPs) to the UNFCCC. Values are central to the design and success of international climate change agreements, yet our empirical understanding of how countries align with one another in common and divergent values – and over time – is severely limited. As we have shown, countries’ core climate change values can be readily recovered from COP HLS speeches. These values can, in turn, be used to understand the structure of negotiation networks at the UNFCCC as well as the shifts in importance of certain values in influencing these networks. In analyzing our resultant value networks for COPs 16–28, we find that initially influential values of “Fairness” and “Power” have increasingly given way to values associated with the “Environment” and “Achievement.” Substantively, this suggests that countries at the UNFCCC have moved away from debates over common but differentiated responsibilities and are increasingly moving towards a consensus over the urgency of collectively and actively combating climate change via mitigation efforts. For policymakers, these insights illustrate the potential of our approach for recovering and understanding countries’ value at UNFCCC negotiations. Given that recognition of negotiating parties’ core values is a necessary first step for any successful climate change cooperation, this paper’s insights accordingly stand to improve the prospects of finding common ground in future climate change negotiations and agreements.

## Figures and Tables

**Figure 8.**
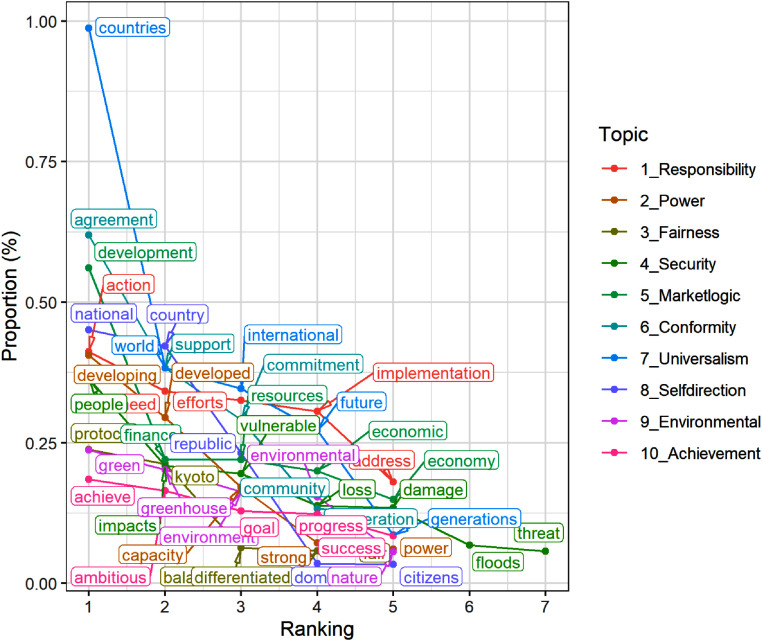
Frequency of keywords by topic. Keyword frequency – as measured by proportion (%) on the *y*-axis – is operationalized as the number of times a keyword appears in our speech corpus, divided by the total length of our speeches. Ranking on the *x*-axis provides the order of within-topic keyword frequency from most to least frequent, which are also presented in Table 6 for interpretability.

**Figure 10.**
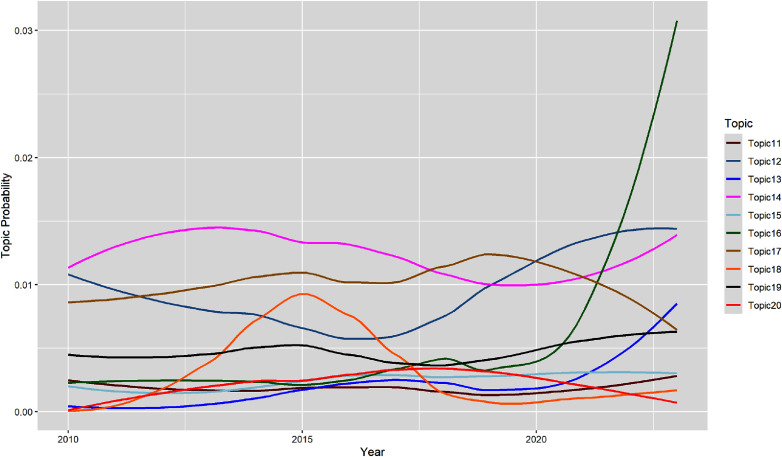
Temporal plots of topic probability for non-keyword topics. Colored lines correspond to averages of each non-keyword topics’ estimated topic probabilities for a particular year (COP), smoothed via a generalized additive model (GAM).

## Data Availability

The data that support the findings of this study are available at Harvard Dataverse (https://dataverse.harvard.edu/ at https://doi.org/10.7910/DVN/AAK1FU).

## Notes

1
With the exception of 2020 due to the COVID-19 pandemic.

2
Drawing on Steg and de Groot (2012), we define values in our international negotiation context as a set of general, transcendent, and abstract beliefs that shape countries’ interests and incentives. While such values are relatively stable over time and not specific to particular negotiation situations, they can evolve and thus reorder countries’ interests and incentives.

3
UNEP: About Loss and Damage: https://www.unep.org/topics/climate-action/loss-and-damage/about-loss-and-damage.

4
In following a grounded theory approach as well as past efforts to categorize similar values, Rosales contends the methodology used to recover these values is consistent with the manner by which values are expressed.

5
One important exception is 2020, which was impacted by the global coronavirus pandemic.

6
While the general public is not accredited to attend COPs sessions without a formal affiliation with one of the above entities, the broader media and organizational staff are two additional sets of actors that are present at COPs.

7
UNFCCC. Party Groupings. https://unfccc.int/process-and-meetings/parties-non-party-stakeholders/parties/party-groupings.

8
Including, for example, “may,” “shall,” “can,” “also,” words such as “1st,” “2nd,” “3rd,” and a small set of non-standard special characters and symbols that arose during the OCRing process.

9
That is, the same d.g.p. as in the case of non-keyword topics.

10
Note that we verify the sensitivity of our results to this choice by estimating models with alternative non-keyword topic numbers in the Supplemental Appendix.

11
As based upon the KeyATM’s token probability scores.

12
Via, e.g., discussion on renewable energy technologies and associated emissions reductions.

13
No COP was held in 2020 due to COVID-19.

14
Five nations have multiple observations within years (Angola, Armenia, Azerbaijan, Ecuador, and Malta) due to having multiple speech opportunities. These are included as separate nodes representing the topic breakdown of the secondary speech.

15
This is consistent with several past studies that stress the centrality of these specific values in international climate change cooperation (e.g., Victor, 2011; Friman and Hjerpe, 2015; Falkner and Buzan, 2022).

16
Thus reinforcing extant insights into the EU’s and European nation-states’ leadership roles in this area and period (e.g., Bueno and Del, 2020; Oberthür and Dupont, 2021).

17
Especially evident at COP 15 in Copenhagen.

18
UNFCCC. Cancun Agreements. https://unfccc.int/process/conferences/pastconferences/cancun-climate-change-conference-november-2010/statements-and-resources/Agreements.

19
For more details, see McNamara (2014).

20
UNFCCC. Warsaw Outcomes. https://unfccc.int/process/conferences/the-big-picture/milestones/outcomes-of-the-warsaw-conference.

21
Center for Climate and Energy Solutions. COP 20 Lima. https://www.c2es.org/content/cop-20-lima/.

22
UNFCCC. Key aspects of the Paris Agreement. https://unfccc.int/most-requested/key-aspects-of-the-paris-agreement.

23
UNFCCC. Key aspects of the Paris Agreement. https://unfccc.int/most-requested/key-aspects-of-the-paris-agreement.

24
Center for Climate and Energy Solutions. COP 22 Marrakesh. https://www.c2es.org/content/cop-22-marrakech/

25
House of Commons Library. What were the outcomes of COP26? https://commonslibrary.parliament.uk/what-were-the-outcomes-of-cop26.

26
UNFCCC. Five Key Takeaways from COP27. https://unfccc.int/process-and-meetings/conferences/sharm-el-sheikh-climate-change-conference-november-2022/five-key-takeaways-from-cop27.

27
UNFCCC. COP28: What Was Achieved and What Happens Next? https://unfccc.int/cop28/5-key-takeaways.

## Appendix A. Web sources for speech texts

Table 5 presents the specific UNFCCCCOP High Level Segment (HLS) website URLs we we utilized when scraping our HLS speech documents.

**Table 5.** Web sources. This table lists presents the specific websites that were used in collecting the COP High Level Segment (HLS) speech transcripts that were used in all subsequent analysis steps

COP	URL
COP 16	https://unfccc.int/process/conferences/pastconferences/cancun-climate-change-conference-november-november-2010/statements-and-resources/cop-16-statements
COP 17	https://unfccc.int/process/conferences/pastconferences/durban-climate-change-conference-november-2011/statements-and-resources/Statements
COP 18	https://unfccc.int/process/conferences/pastconferences/doha-climate-change-conference-november-2012/statements-and-resources/statements-delivered-at-high-level-segment-of-cop-18--cmp-8
COP 19	https://unfccc.int/process/conferences/pastconferences/warsaw-climate-change-conference-november-2013/statements-and-resources/high-level-segment-statements-by-heads-of-states-and-governments- and-ngos-at-cop-19-cmp-9
COP 20	https://unfccc.int/process/conferences/pastconferences/lima-climate-change-conference-december-2014/statements-and-resources/high-level-segment-statements-by-heads-of-states-and-governments- and-igos-and-ngos-at-cop-20-cmp-10
COP 21	https://unfccc.int/process/conferences/pastconferences/paris-climate-change-conference-november-2015/statements-and-resources/High-Level-Segment-statements-COP-21-CMP-11
COP 22	https://unfccc.int/statements-made-during-joint-high-level-segment-of-cop-22/cmp-12/cma-1
COP 23	https://unfccc.int/process-and-meetings/conferences/past-conferences/un-climate-change-conference-november-2017/events-and-schedules/high-level-segment/high-level-segment-statements- of-cop23/cmp13/cma12
COP 24	https://unfccc.int/process-and-meetings/conferences/past-conferences/katowice-climate-change-conference-december-2018/events-and-schedules/high-level-segment/high-level-segment-statements
COP 25	https://unfccc.int/process-and-meetings/conferences/past-conferences/un-climate-change-conference-december-2019/speeches-and-statements-at-cop-25
COP 26	https://unfccc.int/cop-26/speeches-and-statements
COP 27	https://unfccc.int/cop27/high-level#Speeches-and-statements
COP 28	https://unfccc.int/cop28/high-level

## Appendix B. Keyword assisted topic model details

In this appendix section, we first present additional details on the keywords assigned to our Keyword Assisted Topic Model (KeyATM). First, Figure 8 presents the frequency of our chosen keywords for each topic, as measured by the number of times a keyword appears in our speech corpus, divided by the total length of our speeches. While it is difficult to fully visualize all keywords on this plot, given the number of keyword-designated topics that we consider, one can readily observe in Figure 8 that the vast majority of chosen keywords occur at frequencies that surpass the recommended 0.1% threshold proposed by Eshima, et al. (2023). Table 6 presents the final ranking of keywords for each topic based upon these frequency values, to aid in the interpretation of the relative frequency of each keyword for our corpus.

**Table 6.** Keywords for KeyATM, sorted by keyword frequency. The most frequent keywords appear in the keyword 1 column, followed by less frequent keywords in descending order. Keyword frequency is measured by the number of times a keyword appears in our speech corpus, divided by the total length of our speeches. It matches the frequencies presented in Figure 8

Value	Keyword 1	Keyword 2	Keyword 3	Keyword 4	Keyword 5	Keyword 6	Keyword 7
Responsibility	Action	Need	Efforts	Implementation	Address		
Power	developing	developed	capacity	strong	power		
Fairness	protocol	kyoto	balanced	differentiated	fair		
Security	people	impacts	vulnerable	loss	damage	floods	threats
Market-logic	development	finance	resources	economic	economy		
Conformity	agreement	support	commitment	community	cooperation		
Universalism	countries	world	international	future	generations		
Self-direction	national	country	republic	domestic	citizens		
Environmental	green	greenhouse	environmental	environment	nature		
Achievement	achieve	ambitious	goal	progress	success		

*Note:* Five keywords used for all topics except for the Security topic, which uses seven keywords.

Next, we present a series of key outputs from our final KeyATM. First, we provide a series of model fit diagnostics in Figure 9. As can be seen in this figure, two primary measures of KeyATM model fit – log-likelihood and perplexity – both stabilize well before our KeyATM’s assigned number of iterations (of 5,000) is reached. Next, we provide tables of our final topics’ top tokens, as based upon our KeyATM’s word-probability estimates in Tables 7–8. While we present detailed interpretations of each topic that draw upon these top tokens further below, we can note at this juncture that all 10 of our keyword-based topics in Table 7 exhibit multiple specified keywords within their top 10 highest probability tokens. Likewise, we find very few instances across the top tokens in Tables 7–8 of a given topic’s keywords appearing within another estimated topic. Together, this suggests that our recovered keyword-based topics are indeed capturing the constructs underlying our chosen keywords and, hence, our intended values.

Finally, to validate the above contentions further, we provide in-depth summaries of each estimated topic below. These summaries are based upon (i) our review of the top tokens associated with each topic, as presented in Tables 7–8, and (ii) qualitative readings of each topic’s top 20 most highly associated speeches – as recovered from our KeyATM.

**Topic 1: Responsibility** In line with our expectations, this first topic primarily intersects with (i) global responsibility in responding to climate change and (ii) the international community’s role in addressing this issue. The most highly-associated speeches with this topic were found to be ones that call for action and reaffirm countries’ determination to fight climate change and/or to reduce emissions levels. Looking over these highly associated speeches, we find that some countries, such as South Korea, emphasize that the international community “need[s] to build on this moment and answer these calls by taking action.” Others, such as a speech by Denmark, claim that countries “now have to prepare – in a spirit of global responsibility.” In a similar vein, developing countries with speeches found to be highly associated with Topic 1, such as Guyana, express solidarity in calling for global actions while stating that “overarching political commitment is needed to bring the elements together and to inspire and scale up actions that can lead to a low carbon future.” Pakistan, on the other hand, emphasizes its responsibility to comply with international standards in one of its highly associated speeches with Topic 1. To this end, it notes that “in line with [its] commitment to support global efforts” it “ratified the Paris Climate Agreement.” The most commonly used tokens within these qualitatively reviewed speeches include “action,” “need,” “global,” “efforts” and others – in line with this topic’s keywords. Likewise, other top tokens for Topic 1 such as “implementation,” “work,” “finance,” and others are also frequently used in the contexts of individual countries describing their efforts to implement agreements and various projects that affirm countries’ responsibilities to act.

**Table 7.** Top 10 tokens for keyword-based topics

1. Responsibility	2. Power	2. Fairness	4. Security	5. Market-logic
Climate	Countries [7]	President	President	Change
Action	developing	kyoto	climate	climate
Need	change	protocol	people	development
Implementation	climate	commitment [6]	change	resources
Global	developed	durban	impacts	economic
Change	adaptation	parties	vulnerable	sustainable
Finance [5]	development [5]	madam	damage	country [8]
Address	support [6]	period	loss	economy
Efforts	convention	cancun	government	measures
Work	technology	conference	adaptation	natural
6. Conformity	7. Universalism	8. Self-direction	9. Environmental	10. Achievement
Agreement	world	climate	energy	climate
Paris	international	national	green	achieve
Parties	countries	republic	emissions	ambitious
President	future	gentlemen	carbon	new
Cooperation	change	ladies	environmental	time
Conference	climate	country	gas	now
Climate	planet	nations	renewable	goal
Change	responsibility	conference	greenhouse	global
Minister	many	change	reduce	make
Government	one	united	transition	emissions

A  denotes a keyword assigned to a topic by the authors during KeyATM estimation A number in brackets denotes a keyword that was originally assigned to another topic.

**Table 8.** Top 10 tokens for non-keyword topics

Topic 11	Topic 12	Topic 13	Topic 14	Topic 15
Pakistan	Islands	Singapore	Conference	Uganda
Belize	Island	asean	convention	Israel
Haiti	small	Kenya	state	Malta
Somalia	president	que	effects	water
President	states	Mongolia	united	Finland
Charcoal	pacific	les	efforts [1]	Czech
Bahamas	sids	Nigeria	framework	use
Government	ocean	los	Sudan	Denmark
Sids	nations	nous	thanks	Hungary
Reality	unfccc	las	projects	committed
Topic 16	Topic 17	Topic 18	Topic 19	Topic 20
United	countries [7]	Armenia	Malaysia	Tonga
Emirates	change	Azerbaijan	Iceland	Australia
Arab	climate	Brazil	climate	pope
Cop28	peoples	South-Sudan	Nepal	see
Canada	president	region	mountain	francis
Dubai	Guatemala	mitigation	glaciers	pacific
Portugal	indigenous	Turkmenistan	forest	human
Liechtenstein	convention	convention	suriname	Australia’s
Georgia	honduras	delegation	Tajikistan	home
Turkey	mother	deforestation	Bhutan	holy

A number in brackets denotes a keyword assigned to a keyword-based topic.

**Topic 2: Power** The most highly associated speeches with Topic 2 primarily revolve around issues of the power imbalance between developing and developed countries with respect to combating climate change challenges. In these speeches, countries often highlight either (i) a need and/or intention to offset power imbalances by increasing developing countries’ access to technological resources or (ii) demands and challenges associated with getting access to such technologies. For example, in one highly associated speech, China commonly expresses its concern about the capacity gap between developed and developing countries and highlights that technology mechanisms should “promote and accelerate the development and transfer of climate technologies, provide support for developing countries to mitigate and adapt to climate change.” East Timor, on the other side, in expressing the severe impact of climate change, notes that “the most vulnerable and the most affected are those who have limited financial, technological and human capacities to adapt to the impacts of climate change.” Other speeches that received high probabilities of association with Topic 2 similarly highlight elements of power imbalances between industrialized and low-income countries and, oftentimes, will, in turn, call for the reduction of such imbalances. For example, a highly associated speech offered by Thailand emphasizes that “capacity-building support for climate action is critical for developing countries” and that such support “should be responsive to national needs”. It further states that sustainable climate financing should be provided to the developing world in an effort to transform their economies in accordance with climate resilience. This position is shared by other countries belonging to the Global South within Topic 2’s most highly associated speeches. In turn, the interpretation outlined above is also clearly reflected in the top tokens associated with Topic 2, including “countries,” “developing,” “developed,” “development,” “support,” “technology,” among others.

**Topic 3: Fairness** Topic 3 broadly relates to the themes of equity. It specifically addresses issues such as the fair distribution of climate finance, equal participation of parties in agreements, fair transfer of technologies, prioritization of the needs of vulnerable countries, ensuring loss and damage mechanisms, and other related themes. This is clearly illustrated with the statements that our KeyATM determined to be most highly associated with Topic 3, which are in large part from developing countries. These speeches, by and large, call for a more just way of addressing the global climate change problem. For example, Algeria notes in one such speech that country leaders should be guided by a high-level political consensus and that “Parties to the UNFCCC and the Kyoto Protocol should protect the climate system on the basis of equity and in accordance with the principle of common but differentiated responsibilities.” Other countries frequently highlight the importance of equity principles in considering low-carbon development pathways for developing countries while expressing their commitment to fairness and equity principles. In one of these instances, China, for example, notes that it “looks forward to working” with the international community “to achieve a comprehensive, fair, and balanced outcome in Doha to enable the full, effective, and sustained implementation of the Convention and its Kyoto Protocol.” In discussing the second commitment period under the Kyoto Protocol, Tajikistan similarly emphasized that mutuality and proportionality should be a priority by stating that it “expect[s] all the developed countries to sign up to the second commitment period […] [and it] also expect[s] all developing countries to sign up.” In line with these examples one can observe top tokens for Topic 3 such as “kyoto,” “protocol,” “parties,” and “commitment” – each of which help to affirm Topic 3’s interpretation in accordance with our fairness label.

**Topic 4: Security** The underlying content of Topic 4 focuses on linkages between different dimensions of security and the effect(s) of climate change. In particular, the speeches with the highest probability scores under Topic 4 engage with the extent to which human security, economic security, and national security are affected by the adverse impacts of (climate change-linked) environmental anomalies. Delegates representing different countries, particularly climate-vulnerable countries, are further found to express concerns about human displacements caused by climate change commonly, the loss of biodiversity (and its negative effects on their economies and the livelihoods of their people), and the impacts of environmental events on the routine functioning of their states. For example, in one highly associated speech, Malawi draws attention to the severity of problems it experiences due to environmental threats. To this end, Malawi stresses in this speech that “[p]eople are dying. People are displaced. Livelihoods are threatened.” It also frequently emphasizes tangible related security costs. For example, the country’s delegate indicated that “damage and losses on account of the floods were estimated at two hundred million United States dollars.” Other developing countries express acute concerns over their (in)security in a similar manner. For example, South Sudan notes in a highly associated speech with Topic 4 that extreme climate events resulted in crop failure that impacted people’s livelihoods. Its country representative then further cautions that “[o]ver 60% of our people are now categorized as food insecure.” Similarly, in relation to severe environmental challenges, Uganda emphasizes in its own highly associated speech that “all these [events] have brought corresponding problems of severe famine and malnutrition for the affected communities.” In sum, the insecurity that climate change poses to developing countries is omnipresent in Topic 4’s most highly associated statements. Yet, given these challenges, countries in this grouping also highlight the adaptation measures that they undertake to mitigate the effects of the threats. To this end, the identified top tokens – such as “vulnerable,” “climate,” “people,” “damage,” “loss”, and “adaptation” – reflect these varied concerns and thus justify our interpretation of the given topic.

**Topic 5: Market Logic** Our review of Topic 5’s most highly associated tokens and speeches suggests that this topic centers on countries’ determination to promote economic growth in a sustainable manner, including in a manner that allows countries to both address climate change and continue to develop. Topic 5’s most highly associated speeches primarily highlight countries’ adaptation steps toward a sustainable future while underscoring the need to avoid causing significant harm to their national economies. In this vein, these speeches also emphasize new technologies that countries are developing, using, and/or selling to other countries as well as the role of domestic institutions that countries have established to address sustainability alongside future development. For example, Tajikistan’s national priorities, according to its highly associated speeches with Topic 5, include “ensuring environmental sustainability and rational management of natural resources.” Angola’s economic priorities in a similar highly associated speech include “protection of animal and plant wildlife and the development of sustainable international tourism.” At the same time, Brazil, a country with one of the largest GDPs in Latin America, claims in its own highly associated speech that “we [the international community] need to change our development path, revise unsustainable patterns of production and consumption, and promote a low-carbon economy in the long run.” Guatemala, on the other hand, underscores its determination to decouple economic growth from GHG emissions by promoting “artisanal systems for cleaning floating debris located in bodies of water” named Biobardas in another highly associated speech with Topic 5. Finally, among other states, Honduras, in one of its highly associated speeches, highlights the country’s efforts to institutionalize climate change issues by creating a Climate Change Division within the Environmental Ministry and adopting “the National Climate Change Strategy, which addresses the problem within the framework of the national vision, matching the economic, social and environmental” needs. Consistent with this discussion our keyATM approach identifies top tokens such as “development,” “climate,” “change,” “economic,” and “sustainability.” These top tokens and others confirm our interpretation of Topic 5 as primarily focusing on sustainable development aspects under the theme of market logic.

**Topic 6: Conformity** Topic 6’s underlying content is less focused than that discussed above because it relates to the overall aspirations of countries in collective efforts to combat climate change. To wit, Topic 6 includes speeches that highlight the importance of consensus, cooperation, and global compliance with the common goal of reducing GHG emissions. In this regard, and according to the most highly associated documents for this topic, HLS speakers frequently call for mutual efforts and worldwide adherence to international agreements such as the Paris Agreement and other compliance mechanisms – in each case with an eye towards effectively addressing the climate threat. For example, one highly associated speech from Peru emphasizes “that it is essential to strengthen cooperation and collaboration between the Parties.” Peru then furthermore stresses that “consensus, speeding up the technical work [is] required to implement the Paris Agreement in a balanced and transparent manner.” Belarus, on a similar note, states in one of its highly associated speeches with Topic 6 that “each country has unrealized potential that can and should be involved in providing timely additional financial and technical support based on multilateral and bilateral cooperation.” In a similar fashion, other countries with highly associated speeches for Topic 6 note that the only way to address the climate problem is to cooperate with others, since it is collective efforts that will produce the most effective outcomes. For example, Thailand highlights the role of the Paris Agreement in observing that “we [the international community] must continue to work in the spirit of inclusiveness, transparency and solidarity and ensure a comprehensive and balanced progress.” Myanmar and Ukraine, among others, also express desires for collective efforts and remark that climate change is causing a serious impact that requires close cooperation by all within their highly associated speeches. To this end, the top tokens for this topic – including “paris,” “agreement,” “cooperation,” and “parties” – together demonstrate these same themes of collective conformity with global efforts to combat climate change.

**Topic 7: Universalism** Topic seven, which we term Universalism, centers on the importance of universal commitments under climate agreements – especially with a priority of saving the planet for future generations. In the speeches of county representatives that we found to be most highly associated with this topic, speakers often stress that global cooperation and solidarity are paramount for addressing common objectives and saving humanity. In these contexts, the content of the statements that we found to be most highly associated with Topic 7 frequently refers to a higher mission projected into the future, where universal commitment and partnership-building are keys to overcoming negotiation deadlock. At the same time, some countries with highly associated Topic 7 speeches express their dissatisfaction with the global promotion of capitalism and go on to claim that it undermines universal efforts to achieve global carbon neutrality. For example, in the context of the climate change threat, Belarus notes that “[w]e talk about the future of our children and grandchildren. We talk about the future of all those coming into this world after us.” By linking current threats with future implications in this manner, country representatives convey concern on a broader scale and, hence, universalism. Similarly, a representative of the Holy See observed that “leaving a habitable planet to future generations is, first and foremost, up to us. Humanity is one family. As brothers and sisters, we have only one home, one common home, and we all must care for it.” On the other hand, Bolivia’s most highly associated speech with Topic 7 (similar to another from Cuba) opines that “you cannot achieve harmony with nature and the well-being of humanity if the same patterns of consumption and production of capitalism are followed, these patterns have condemned our Mother Earth to death.” In these manners, we conclude that speeches associated with Topic 7 demonstrate that – in the words of Macedonia’s country representative – “‘climate change is a global phenomenon which requires a global response of the global community that believes in this mission.” This clearly captures a universal approach to addressing climate change. This value and overall interpretation are each also reflected in the top tokens for Topic 7, most notably “world,” “future,” “planet,” and “countries,” among others.

**Topic 8: Self-direction** Topic 8 stands out among the previously discussed topics for its focus on countries’ individual efforts to address climate change problems rather than global cooperation. More specifically, in the statements with the highest association with Topic 8, country leaders frequently outline the climate mitigation or adaptation measures that they have taken or plan to undertake at the national level. For example, in one highly associated speech, Mali reports that it “adopted a Strategic Framework for a Climate-Resilient Green Economy [and] a Nationally Determined Contribution (NDC),” in which it “commits to an average reduction of 40% of greenhouse gas emissions by 2030 with 13 new programs relating to renewable energies, sustainable land, and waste management, agricultural and forestry sectors and value chains.” Similarly, a Burundi delegate notes in another highly associated speech that the “government of Burundi has the Climate Change Policy, Strategy and Action Plan, the recently revised Forest Code of 2016, the Climate Change Vulnerability Study and a communication strategy on climate change.” Other countries with highly associated Topic 8 speeches similarly focus on discussing their attempts to respond to climate change within their national capacities. As another example, Niger’s leader, among the other countries’ representatives, observed in one highly associated speech that their country implemented the “Niger-2035 Sustainable Development and Inclusive Growth Strategy, the Plan de Economic and Social Development (2017–2021), and the Nationally Determined Contribution.” In this sense, while many highly associated speeches continue to emphasize the overall challenges of and demand for international cooperation, they share a primary similarity in their levels of emphasis on information about national actions to prevent or respond to the global climate threat. This value, furthermore, is also captured within the top tokens presented above. Examples include “climate,” “national,” “republic,” and “country,” among others.

**Topic 9: Environmental** The top-level environmental issue underlying Topic 9 appears to be concern over greenhouse gas emissions. While this focus is more narrow than the full range of climate change-associated environmental concerns, it is also understandable given (i) the focus of this forum and (ii) the fact that other topics, such as Security, already cover other environmental dimensions of global climate change. Beyond outlining concerns over greenhouse gas emissions, a significant share of the most highly associated speeches with the Environmental topic convey countries’ efforts and/or plans to achieve major emissions reductions in light of the contributions of greenhouse gases to climate change. This includes efforts to directly reduce emissions or the pursuit of steps to achieve this goal, such as green or renewable mechanisms. For example, Kazakhstan discusses the importance of green finance, green growth, and the green economy in one highly associated speech. Japan likewise reports that it “intends to achieve maximum deployment of clean energy through making renewable energy the main source of power.” Lithuania, in a similar manner, indicates that it “will finalize the National Long-term Low Carbon Development and Adaptation Strategy with a view to reaching net-zero emissions by 2050.” Denmark also conveys strong goals in this vein by expressing that it will “become independent of fossil fuels by 2050.” This is similar to a highly associated speech offered by Russia, which claimed that it “adopted a new 2050 Strategy of Social and Economic Development with Low Greenhouse Gas Emission,” which promises to bring the “country to an 80% net reduction of greenhouse gas emissions while maximizing absorbing capacity of our forests and other ecosystems.” Other countries have similar claims and set ambitious goals for the future, which leads us to interpret this topic as encompassing Environmental values from the standpoint of addressing the greenhouse gas-related sources of the climate change problem. This interpretation is also evident within the top tokens for Topic 9, including “green,” “energy,” “environmental,” “renewable,” and “reduce,” among others.

**Topic 10: Achievement** Topic 10, which we title Achievement, focuses on three interrelated components to this particular value: (i) achievements made in the past by countries, (ii) current achievements, and (iii) future achievements that countries pledge to attain. In this regard, while some of the speeches and themes under this topic overlap with environmental values and conformity values in the context of achievements, the main thrust of this topic centers on countries’ expressions of the ultimate target or achievement associated with these efforts. For example, in one of the most highly associated speeches with Topic 10, Germany observes that it “not only met its Kyoto targets, but it has also exceeded them” and that the country has already “achieved a 26% reduction in our greenhouse gas emissions compared with 1990” despite its economic growth. In this manner, Germany’s leader highlights that a broader goal of fighting climate change (which also intersects with Topic 9: Environmental) has been achieved to some extent. Similarly, Estonia notes in a separate highly associated speech that it “has managed to cut its CO2 emissions by half compared to 1990.” In terms of the second component noted above – current achievements – some countries emphasize their active or ongoing efforts as a real-time achievement of sorts. For example, Malta reports in its own highly associated speech that “[b]eing an EU Member State, we are working hard to achieve a 55% emission reduction by 2030 from the 1990 levels. Malta has achieved a lot during the past years, but we want to be more ambitious and do much more.” Similarly, a highly associated speech from the Netherlands remarks that “I won’t deny that it’s a painstaking and costly process, but the good news is: it can be done. We’re investing in large-scale renewable energy, with a special focus on green hydrogen both in the Netherlands and abroad.” In the context of future achievements, several highly associated speeches see countries projecting necessary steps for securing future achievements. Switzerland, for example, discusses the importance of maintaining its position as a financial center that aligns with the goals of the Paris Agreement in another highly associated speech. To achieve this objective, the country notes that it is crucial to implement strict regulations and ensure the agreement’s effective implementation in the future. Based upon these interpretations, we accordingly see this topic’s top tokens (e.g., “climate,” “achieve,” “goal,” and “emissions”) to fit within our notion of the achievement value.

**Top** **ic** **11** Topic 11’s top tokens are less informative than our earlier topics. Even so, the most highly associated speeches for Topic 11 at times capture speeches offered by a small set of countries facing severe impacts of climate change. More specifically, as the top tokens associated with this topic were identified, countries such as Haiti, Belize, and Somalia each face severe challenges due to climate events and/or internal instabilities. To this end, we find, for example, that Somalia’s speeches often discuss the extent to which the effects of civil war and corruption have led to overexploitation of its terrestrial and marine resources. The country’s representative notes in one case, for example, that “corrupt Somali merchants in cahoots with the businessmen in the countries that import [Somali] charcoal are responsible for the depletion of the mangrove forests in Somalia that play a critical role in mitigating climate change.” In light of this, and the potential for such challenges to be aggravated by the climate events related to floods, drought, and loss of biodiversity, Somalia then goes on to claim that it does not receive the attention it deserves at the international stage. Haiti likewise suffers from the adverse effects of climate change and development challenges, and also faces frequent environmental anomalies such as hurricanes. In light of this, a Haiti representative claimed in one highly associated speech that “[t]he increase in the number and intensity of extreme weather events is causing considerable damage to the country’s economy, affecting people’s way of life and causing extreme poverty.” Belize suffers from similar climate change challenges. And Haiti is losing its barrier reef very rapidly, which affects the country’s economy, culture, and tourism, which “contributed approximately 40% to the country’s GDP.” The country’s representatives often highlight the losses due to coral bleaching stress and alert the international community to these issues. In sum, this topic appears to be capturing a specific aspect of extreme vulnerability to climate change that is likely not shared by many of the countries and speeches that are captured within the more general Security (value) Topic discussed above.

**Topic 12** Topic 12 primarily encompasses speeches by representatives from small island states. These states are uniquely vulnerable to the adverse effects of climate change on sea level rise. Countries such as Palau, Marshall Islands, Tuvalu, Fiji, and the Maldives, among others, are the most common countries expressing concern about their survival that depends on international efforts to fight climate change. In the most highly associated speeches for Topic 12, representatives of these countries raised particular concern over international (in)actions pertaining to the mitigation of climate change’s adverse effects. For example, a Maldives representative stated that “small island states are a special category of nations, nations that have done little or nothing to cause the climate crisis but who stand to be hit first and hit hardest. These nations need particular adaptation support.” Similarly, Tuvalu’s representatives expressed concerns over the immediate effects of climate change for states such as Tuvalu at virtually every COP considered. In one case, Tuvalu’s leader indicated that “Tuvalu and other small island nations come to COP meetings to remind the world, especially the biggest emitters of greenhouse gases, of our relentless suffering.” Palau’s leader, like many other leaders of small island states, expressed similar concerns and stated in one instance that “[o]ur resources are disappearing before our eyes, our future is being robbed from us […] you might as well bomb our islands instead of making us suffer only to witness our slow and fateful demise.” To this end, the top tokens for this topic – such as “islands,” “small,” “pacific,” and others – help to confirm that this topic is primarily focused on small island states’ specific anxieties surrounding climate change’s effects.

**Topic 13** Topic 13 is less consistent than the others. This is reflected in its top tokens, which contain a small number of non-English phrases such as – “nous”, “las”, “les”^28^ – alongside top tokens corresponding to specific countries such as “Kenya,” “Singapore,” and “Mongolia.” The latter countries are often the countries whose speeches are identified under Topic 13 with high probability. Hence, this topic appears to be capturing unique qualities of these countries, potentially alongside opening and closing statements to our HLS speeches more generally. Regarding of the unique qualities of the countries included, these Asian and African countries are generally vulnerable to climate change but constrained in their contributions to the problem, which may be driving this commonality. From the speeches of these countries’ representatives, we can observe clear expressions of concern for the future alongside calls for action by the international community. For example, Singapore’s leader, after raising concern over extreme weather events, stated in one recent COP that “Parties need to show that they are delivering real emission reductions that align with their NDCs.” Mongolia’s representative similarly noted that “I would like to appeal to all Parties to decisively take concrete actions with a more ambitious climate target to keep our planet for generations to come and to ensure the safety of our co-habitants.” Kenya and Nigeria instead appear to be mostly concerned with capacity-building mechanisms and support for addressing challenges such as poverty and nutrition security in their countries. Overall, this topic appears to be capturing a broad range of concerns held by developing countries, generally in relation to the effectiveness of climate change negotiation outcomes.

**Topic 14** The most highly associated speeches with Topic 14 primarily include speeches made by countries in the Middle East and North Africa (MENA) region. In their speeches, the leaders of these countries emphasize the importance of the COPs and internationally binding conventions, reaffirm their commitments, and highlight the importance of common efforts in addressing the climate problem – in addition to highlighting their own unique problems. For example, Kuwait frequently highlights the role of international cooperation and the significance of the conferences. Kuwait’s country representative at one COP noted in this regard that “It is our pleasure to see this high-level international participation in this conference, which reflects the great importance that the international community attaches to the issue of climate change.” Oman, in a similar manner, highlights the central role of collective efforts in stressing the importance of “transparency emphasized by both the United Nations Framework Convention on Climate Change and the Paris Agreement on climate change.” Based upon these examples and others, this topic appears to primarily be about the UNFCCC itself. This is further evidenced by top tokens such as “conference,” “convention,” “united,” and “framework.” Likewise, and as is the case for other countries, MENA countries often thank the international community for the efforts and appeal to the host country with gratitude for the conference arrangements within their speeches. And it appears this topic is intersecting with this component of our speeches as well, as indicated by top tokens such as “efforts,” “thanks,” and “state.”

**Topic 15** As was the case for some of the earlier non-keyword-assisted topics mentioned earlier, Topic 15 is another topic that is not particularly coherent in a thematic sense. To this end, one can observe top tokens that span a number of European countries, but also Israel and Uganda. This is also evident from the tokens extracted for Topic 15 that primarily consist of country names. Topic 15’s most highly associated speeches mirror this heterogeneity in associated countries. Given the diversity of countries under the topic, their positions and values as expressed in these speeches also somewhat differ. For example, while Uganda is primarily concerned about the effects of climate change on agriculture and the country’s future development prospects, Denmark tends to focus on strategies for achieving green energy and electricity generation within its territory. For example, a Denmark representative noted within one highly associated speech that “‘Denmark’s story is that we have made green energy cheaper than fossil energy. We have come far. And we are going full steam ahead.” While San Marino’s most highly associated speeches with Topic 15 emphasize sustainable development achievements, the highly associated speeches made by Lebanon primarily express worries over forest fires, water shortages, and temperature variations in relation to people’s livelihoods. For example, a Lebanese representative in one instance noted “[w]e have placed among our priorities the cultivation of wheat and the expansion of the wonderful areas, as well as the increase of ponds to collect winter rains.” Overall, Topic 15 can accordingly be seen to include speeches across a wide range of development levels and from different parts of the world, but without much coherence in a thematic sense.

**Topic 16** Topic 16, as evident from its top tokens (such as “united,” “arab,” “dubai,” and “cop28”) primarily encompasses speeches given by various countries at the COP 28 in the UAE. In the most highly associated speeches for this topic, countries appeal to the host country and express their gratitude to the President of the UAE before focusing on their varied positions. This largely explains why the top tokens for this particular topic (outside of those mentioned above) span a wide range of countries across the globe. Countries such as Namibia, Tonga, Lichtenstein, Monaco, and Zambia are among the countries whose speeches are identified as being most highly associated with this topic. While there thematic pattern aside from the COP 28-dimension, it is evident that speeches made by country representatives at this COP tend to stress the importance of this particular COP in light of slow progress in addressing the climate threat during recent years. For example, Lichtenstein asserted in its highly associated speech that the presence of many states at this venue was a good indication, before emphasizing that the international community is “off track and urgent action is necessary in order to adjust” the course of actions. Namibia, similarly, claimed that “COP28 provid[ed] a platform for nations to strengthen partnerships,” while Canada warned that “outside [the COP’s] walls, people are watching COP 28 with both hope and some skepticism.” For this topic’s most highly associated speeches, countries also tended to emphasize the necessity of scaling up the impact of collective efforts in light of the increasing urgency of the climate change threat. Accordingly, this topic appears to loosely focus on speeches that emphasize the uniqueness of COP 28.

**Topic 17** Interestingly, Topic 17 appears to primarily encompass issues specific to Latin American countries. As such, statements from countries such as Venezuela, Bolivia, Guatemala, Nicaragua, and others are found to be among the most highly associated with this particular topic. This regional pattern is at least partly attributable to the fact that Latin American countries at times give speeches on behalf of, or self-affiliate with, the Latin American region as a Group of Latin American and Caribbean States (GRULAC) or other similar regional organizations. In these cases and others, the most highly associated speeches in this topic accordingly tend to emphasize common problems that climate change poses for this region. Alongside this, certain individual countries, such as Bolivia, take certain value-oriented positions on international politics when blaming the existing system for its “selfishness, greed, and unsustainable production and consumption patterns” and a lack of “respect for the life of human beings and Mother Earth.” Nicaragua similarly emphasizes that “it is urgent that, to preserve and defend the Right to Life on our Mother Earth.” This emphasis on respect for the Earth appears to be a fairly distinct characteristic of Latin American countries within our speech corpus. In this vein, these countries tend to underscore the significance of Latin America’s significant forests and the threats facing these forests. For example, Venezuela emphasized that its forests “represent[s] 1.19% of the planet’s forest cover, and 5.13% of Latin America,” which in turn plays a vital role in carbon sequestration. Honduras, also admitting the importance of forests, noted that it suffers from drought and other extreme weather events which together destroy its biodiversity and the livelihood of indigenous people. Other countries, in a similar manner, attempt to alert global leaders to the danger of climate change. Hence, as reflected in the top tokens reported in Table 8 (e.g., “climate,” “peoples,” “mother,” “guatemala,” and “honduras”), this topic primarily encompasses Latin American country concerns alongside an overlapping set of broader forest-oriented concerns.

**Topic 18** Topic 18 encompasses several different themes, including conflicts and instability, desires for sustainable development, and the danger of climate change threats to countries’ landscapes. Many of the most highly associated speeches for Topic 18 focus on Armenia and Azerbaijan. These two countries have used the COPs to accuse each other of environmental problems stemming from their conflict over the Karabakh region. For example, Azerbaijan noted that “challenges for the implementation of relevant reduction targets on greenhouse gas emissions, as well as the efficient realization of climate change mitigation and adaptation actions, are conditioned by the ability of Azerbaijan to exercise effective control over its territory illegally occupied currently by Armenia.” Armenia, in return, shifted the blame to Azerbaijan in its own highly associated speeches, often emphasizing that the latter was responsible for mass killings in the region. On the other hand, South Sudan, whose speeches are also among the most highly associated speeches for this topic, tends to discuss the crop failures and flooding in relation to the threats that these pose to South Sudanese people, while also expressing hope for financial support for various initiatives aimed at combating deforestation. Brazil’s highly associated speeches for this topic in turn appear to express concern with climate change threats, to pledge its intention and achievements in reducing carbon emissions, to discuss the elimination of illegal deforestation, and to pose transitions to renewable energy generation. Given these varied these, the top tokens for Topic 15 appear to be varied and at times country-focused (e.g., “armenia,” “azerbaijan,” “brazil,” and “deforestation”).

**Topic 19** Topic 19’s most highly associated speeches are, by and large, focused on the somewhat unique challenges that arise in Asia and Europe as a result of climate change. More specifically, the common theme among Asian countries such as Kyrgyzstan, Nepal, Bhutan, and Tajikistan is the effects of global warming on mountains and glaciers. For example, in one highly associated speech, Bhutan’s representative noted that the country is small and landlocked with a “fragile mountain ecosystem.” Butan’s representative then notes that “[i]t is highly vulnerable to catastrophic glacial lake outburst floods, drying water sources, increasing landslides and flash floods, freak windstorms, decreasing snowfall, and unpredictable rainfall patterns to name just some of the adverse impacts of climate change.” Similarly, Tajikistan’s leader emphasized in another highly associated speech that Tajikistan is a beautiful “landlocked mountainous developing country with thousands of glaciers, lakes, and rivers,” before conceding that it faces “enormous challenges due to the accelerated melting of glaciers.” European countries such as Norway and Iceland are also featured under this topic out of concern over rising temperatures. For Norway, the Arctic is a primary concern because the melting of ice and snow impacts its indigenous peoples and threatens their existence. In one COP, for example, their country representative noted that “[i]n the Arctic, glaciers are melting at a record pace. In all regions, ecosystems and local communities are at risk.” Iceland, on the other hand, at various COPs reaffirmed its commitment to the rapid reduction of emissions by adopting renewable strategies. Other countries in Asia and Europe linked to this topic also express concerns about deforestation while being primarily focused on glacier melting. Yet in some, this topic appears to primarily be about glacial melting and related climate impacts. These observations are well supported by the top tokens for Topic 19, including tokens such as “climate,” “mountain,” and “glaciers.”

**Topic 20** Topic 20 primarily focuses on focused the Pope’s sovereign jurisdiction (i.e., the Holy See) alongside two other countries: Tonga and Australia. This characterization is also reflected in several of Topic 10’s top tokens, such as “holy,” “see,” “pope,” “australia,” and “tonga.” In their speeches, representatives of the Holy See often emphasize the moral and ethical role of the international community in fighting climate change while calling for reconsideration of current global lifestyle patterns. For example, in one COP, a highly associated Holy See speech indicated that “[i]t is also essential to take into account the ethical aspects of the new development paradigm. Here we enter into the fundamental areas of education and the promotion of lifestyles based on a responsible conscience towards our common home.” A call for global unity is also made by Tonga and Australia, yet more in a pragmatic way. For example, Tonga’s leader warning about the eroding capacities for resilience among small island states as a result of climate change before noting that “the implementation of the Paris Agreement brings new hope. A hope that rests on the conscience of the global citizens.” Australia, on the other hand, while also noting threats to its biodiversity resulting from global warming and sea level rise, often highlights its (domestic and global) contributions and initiatives pertaining to the mitigation of climate change’s adverse effects. For example, in one such highly associated speech, Australia’s representative noted that “Australia’s $16 million Indo-Pacific Land Action Package will help countries strengthen land management, fund public–private sector partnerships, and achieve greater coordination through the NDC Partnership launched in Marrakesh.” As is evident, Topic 20 focuses on different themes that relate to different countries’ priorities and concerns, albeit with some emphasis on themes of unity or hope. This is reflected, to an extent, in top tokens such as “home” and “human.”

## Appendix C. Temporal stability of non-keyword topics

Given the non-keyword topic interpretations above, we next evaluate the extent to which our non-keyword topics are temporally concentrated (i.e., by year or COP) via Figure 10. To create this figure, we calculate each non-keyword topic’s average topic probability across all speeches for each COP. We then plot these temporal trends across all COPs (i.e., years 2010–2023) using generalized additive model (GAM) smoothing. As can be seen in Figure 10, a majority of our non-keyword topics exhibit temporal persistence all COPs considered. Three exceptions are as follows. First, speeches aligned with Topic 18 are largely concentrated in 2015 (COP 21). As we note further above, this topic largely reflected a temporally and substantively unique subset of non-climate change-related content that spilled over into the UNFCCC at this juncture (namely, Nagorno-Karabakh tensions). Second, Topics 13 and 16 both appear to be largely concentrated in 2023 (COP 28). One element of these findings is unsurprising, seeing as Topic 16 – as discussed further above – was primarily related to speeches pertaining to COP 28 and its host, specifically. The same may hold true for Topic 13. However, Figure 10 indicates that this topic is simply not particularly prominent overall and – as noted earlier – it is also less thematically coherent than other non-keyword topics. This altogether limits its interpretability in terms of its increased attention in 2023, though it may reflect at least some distinct developing country concerns that more uniquely began to arise at this juncture.

## Appendix D. Stability of keyword-based topics

Taking a cue from robustness assessments that have been proposed or implemented within past topic model research in the social sciences (Wilkerson and Casas, 2017; Berliner et al., 2020, 242), we next assess the stability of our Keyword-based topics when the number of non-keyword-based topics is modified from its originally assigned number of 10 to either 9 (Tables 9–10) or 11 (Tables 11–12). Together, these alternative specifications allow us to evaluate the degree to which our Keyword-based topic findings are sensitive to minor changes in the total number of – and total number of non-keyword-based – topics estimated. To assess this, we primarily focus on the topwords obtained for both the keywords-based topics and non-keywords-based topics under each scenario, comparing these to our originally obtained topwords in Tables 7–8.

We begin by comparing our keywords-based topics across our original (10 non-keyword) and alternate (9 and 11 non-keyword) topic models. As can be seen across Tables 7, 9, and 11, our keyword-based topics are remarkably stable in their topwords no matter the choice of non-keyword topics considered here. Indeed, although some topwords change slightly in order within specific topics, each each keyword-based topic in Tables 9–11 tends to retain a majority of the topwords originally reported in Table 7. To this end, we can note that the topwords in Table 7 included 33 instances of an assigned keyword to a particular topic appearing in that topic’s top 10 words. By comparison, our 9 non-keyword KeyATM (Table 9) and our 11 non-keyword KeyATM (Table 11) saw 32 and 36 such instances, respectively. The number of topic topwords that contained a keyword originally assigned to another topic was also comparable across our three models, at rates of six (Table 7), seven (Table 9), and five (Table 11).

Direct comparisons of the non-keyword-based topics in Tables 8, 10, and 12 are more difficult to make given that (i) each table contains a different total number of topics and (ii) conceptually similar topics are not always assigned to the same non-keyword topic number across these tables. Even so, one can note a number of key similarities across these non-keywords topics. For instance, Topic 12 in Table 8 lines up very closely with Topic 18 in Table 10 and to a lesser extent with Topics 13 and 19 in Table 12. Similar patterns can be observed across a majority of the additional non-keyword-based topics across these three tables, where the topwords in Table 12 do at times appear to be slightly less interpretable. Even so, the number of keyword terms assigned to our non-keyword-based topics appears consistent across these three choices of non-keyword topic number, with two, three, and five such instances appearing in Tables 8, 10, and 12. Hence, altogether, our primary (Keyword-based) topic estimates in Tables 7 and 8 are not sensitive to small perturbations in our total, and non-keyword-based, number of estimated topics.

**Table 9.** Top 10 tokens for keyword-based topics, when using 9 non-keyword-based topics

1. Responsibility	2. Power	2. Fairness	4. Security	5. Market-logic
Climate	Countries [7]	President	President	Economic
Action	developing	protocol	climate	resources
Change	change	kyoto	change	development
Global	climate	parties	people	water
Need	developed	durban	impacts	natural
President	development [5]	madam	loss	sustainable
Address	adaptation	cancun	vulnerable	region
Eforts	mitigation	period	damage	production
Implementation	capacity	commitment [6]	government	system
Towards	technology	conference	adaptation	forests
6. Conformity	7. Universalism	8. Self-direction	9. Environmental	10. Achievement
Agreement	countries	climate	energy	world [7]
Paris	future	change	emissions	time
Parties	climate	country	greenhouse	now
Climate	change	national	green	new
Conference	world	development [5]	carbon	goal
Community	international	republic	environmental	global
President	one	government	gas	year
National [8]	planet	framework	renewable	progress
Implementation [1]	responsibility	sustainable	climate	years
Minister	today	convention	new	need

A  denotes a keyword assigned to a topic by the authors during KeyATM estimation A number in brackets denotes a keyword that was originally assigned to another topic.

**Table 10.** Top 10 tokens for non-keyword topics, when using 9 non-keyword-based topics

Topic 11	Topic 12	Topic 13	Topic 14	Topic 15
Singapore	Canada	Gentlemen	Conference	Islands
Asean	belize	ladies	convention	president
Liberia	honduras	african	state	pacific
Georgia	Namibia	Africa	united	people [4]
Que	Korea	president	nations	Tuvalu
Redd	change	conference	effects	Tonga
Papua	indigenous	united	excellencies	earth
New-guinea	peoples	excellencies	kingdom	Afghanistan
Hungary	niue	nations	efforts [1]	mother
Los	Peru	climate	framework	peoples
Topic 16	Topic 17	Topic 18	Topic 19	
Climate	climate	small	Kenya	
European	united	Island	Uganda	
Portugal	arab	haiti	Australia	
Croatia	glasgow	Island	suriname	
Ireland	world [7]	vanuatu	les	
Ladies	emirates	sids	bahamas	
Austria	cop26	states	ecuador	
Brazil	Paris	Nepal	Australia’s	
Finland	cop	pacific	des	
Union	cop28	dominica	nous	

A number in brackets denotes a keyword assigned to a keyword-based topic.

**Table 11.** Top 10 tokens for keyword-based topics, when using 11 non-keyword-based topics

1. Responsibility	2. Power	2. Fairness	4. Security	5. Market-logic
Climate	President	Protocol	Climate	Climate
Global	clamate	kyoto	people	change
Action	change	president	change	development
Need	adaptation	durban	president	national [8]
Implementation	capacity	parties	impacts	economic
Address	developing	madam	vulnerable	sustainable
Efforts	developed	period	loss	adaptation
Thank	mitigation	cancun	damage	economy
Ambition	support [6]	convention	island	international [7]
Work	parties	commitment [6]	small	resources
6. Conformity	7. Universalism	8. Self-direction	9. Environmental	10. Achievement
Agreement	countries	climate	energy	time
Paris	world	country	emissions	ambitious
Parties	change	republic	green	now
Community	future	gentlemen	environmental	one
Change	climate	ladies	greenhouse	make
Conference	international	change	carbon	new
Support	developing [2]	conference	renewable	let
Climate	responsibility	president	gas	years
Cooperation	human	united	environment	progress
Commitment	issue	nations	reduce	just

A  denotes a keyword assigned to a topic by the authors during KeyATM estimation A number in brackets denotes a keyword that was originally assigned to another topic.

**Table 12.** Top 10 tokens for non-keyword topics, when using 11 non-keyword-based topics

Topic 11	Topic 12	Topic 13	Topic 14	Topic 15	Topic 16
Sudan	Africa	United	Climate	Mauritius	Peoples
Haiti	Kenya	Emirates	Singapore	que	convention
Chad	African	Arab	Asean	Sierra	Guatemala
Kuwait	Namibia	cop28	Australia	leone	indigenous
Gabon	parties	Dubai	energy	les	earth
Emissions	Malta	Uganda	emissions	monaco	mother
Kyrgyzstan	niger	Tuvalu	Austria	los	honduras
Montenegro	Brazil	Islands	cent	marino	Nicaragua
Sultanate	cameroon	nations	brunei	albania	life
Excellencies	convention	marshall	sector	san	president
Topic 17	Topic 18	Topic 19	Topic 20	Topic 21	
Pakistan	convention	Islands	glasgow	Ukraine	
Malaysia	conference	president	Oaris	Armenia	
Afghanistan	state	liberia	cop26	Azerbaijan	
Nepal	effects	Iceland	Japan	Russian	
Water	efforts [1]	redd	finance [5]	liechtenstein	
Bhutan	framework	solomon	emissions	republic [8]	
Glaciers	gentlemen	palau	united	federation	
Tajikistan	united	dominica	kingdom	Russia	
Madam	support [6]	Papua	support [6]	d’ivoire	
Forest	nations	New-Guinea	Spain	united	

A number in brackets denotes a keyword assigned to a keyword-based topic.
